# Exploring the Impact of Linkage Structure in Ferroelectric Nematic and Smectic Liquid Crystals

**DOI:** 10.1021/acs.jpclett.3c03492

**Published:** 2024-04-10

**Authors:** Hiroyuki Matsukizono, Yusuke Sakamoto, Yasushi Okumura, Hirotsugu Kikuchi

**Affiliations:** † Kyushu University, Institute for Materials Chemistry and Engineering, 6-1 Kasuga-Koen, Kasuga, Fukuoka 816-8580, Japan; ‡ Kyushu University, Interdisciplinary Graduate School of Engineering Sciences, 6-1 Kasuga-Koen, Kasuga, Fukuoka 816-8580, Japan

## Abstract

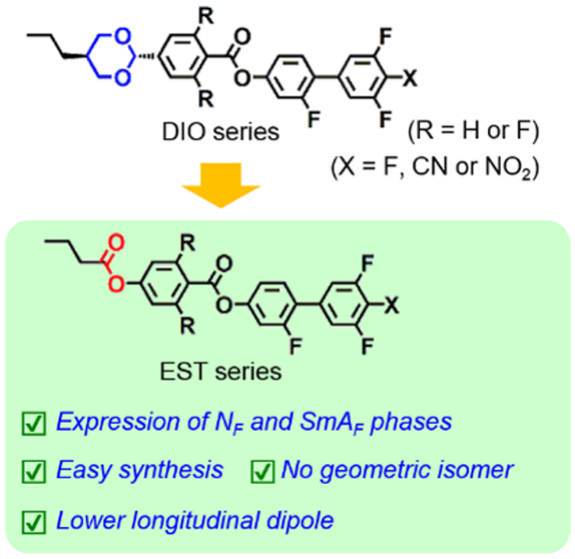

The liquid crystal molecule 3-fluoro-4-(3,4,5-trifluorophenyl)phenyl 2,6-difluoro-4-(*trans*-5-n-propyl-1,3-dioxane-2-yl)benzoate (DIO) has attracted considerable interest owing to its unique ferroelectric nematic phase and extraordinarily high dielectric constant. To expand the DIO series, novel analogs with 1,3-dioxane units converted to ester units (EST analogs) were synthesized, and their physical properties were characterized. The EST analogs exhibited ferroelectric phases similar to those of the corresponding DIO analogs. Interestingly, an EST analogue featuring a defluorinated benzoate unit exhibited a ferroelectric smectic A phase, despite its smaller longitudinal dipole moment of 6.9 D. This result diverges from the common knowledge that the formation of large longitudinal dipoles is traditionally effective in the emergence of ferroelectric phases. Unlike the DIO series, the EST analogs can be readily obtained without the formation of undesired geometric isomers, which is advantageous for practical applications. The results of this study provide valuable insights into the design of liquid-crystalline materials expressing ferroelectric phases.

Liquid crystal (LC) materials have been widely used in industry,^1^ particularly those exhibiting ferroelectric properties owing to their versatility for potential applications,^2^ such as capacitors, energy-conversion materials, and electro-optical devices. Numerous LC materials primarily use LCs exhibiting the nematic (N) phase owing to their quick and easy response to external stimuli in various applications.^3^ Unlike the ordinary N phase, in the ferroelectric nematic (N_F_) phase, dipole moments of anisotropic LC molecules are assumed to align in the same direction along the director. Consequently, the magnitude of the dipole moments combines to generate a macroscopic polarization with a large dielectric constant.^4^ Although LC molecules with an N_F_ phase have been known for a long time, we recently described how a fluorine-based LC composed of a 1,3-dioxane unit, namely, 3-fluoro-4-(3,4,5-trifluorophenyl)phenyl 2,6-difluoro-4-(*trans*-5-n-propyl-1,3,dioxane-2-yl)benzoate (DIO-1, Figure 1) could form an N_F_ phase (referred to as the MP phase in the original study) with an extraordinarily high dielectric constant of more than 10 000.^5^ Concurrently, Mandle et al. reported an unidentified N phase of RM734,^6^ whose ferroelectric features were later confirmed.^7^ Furthermore, an LC molecule showing an N_F_ phase at ambient temperature was recently described by Manabe et al.^8^ Although ferroelectric LC materials have been extensively studied recently,^9−20^ the origin of ferroelectricity, including the propagation of the microscopic orientation of molecular dipole moments to macroscopic spontaneous polarization, remains poorly understood.

**Figure 1:**
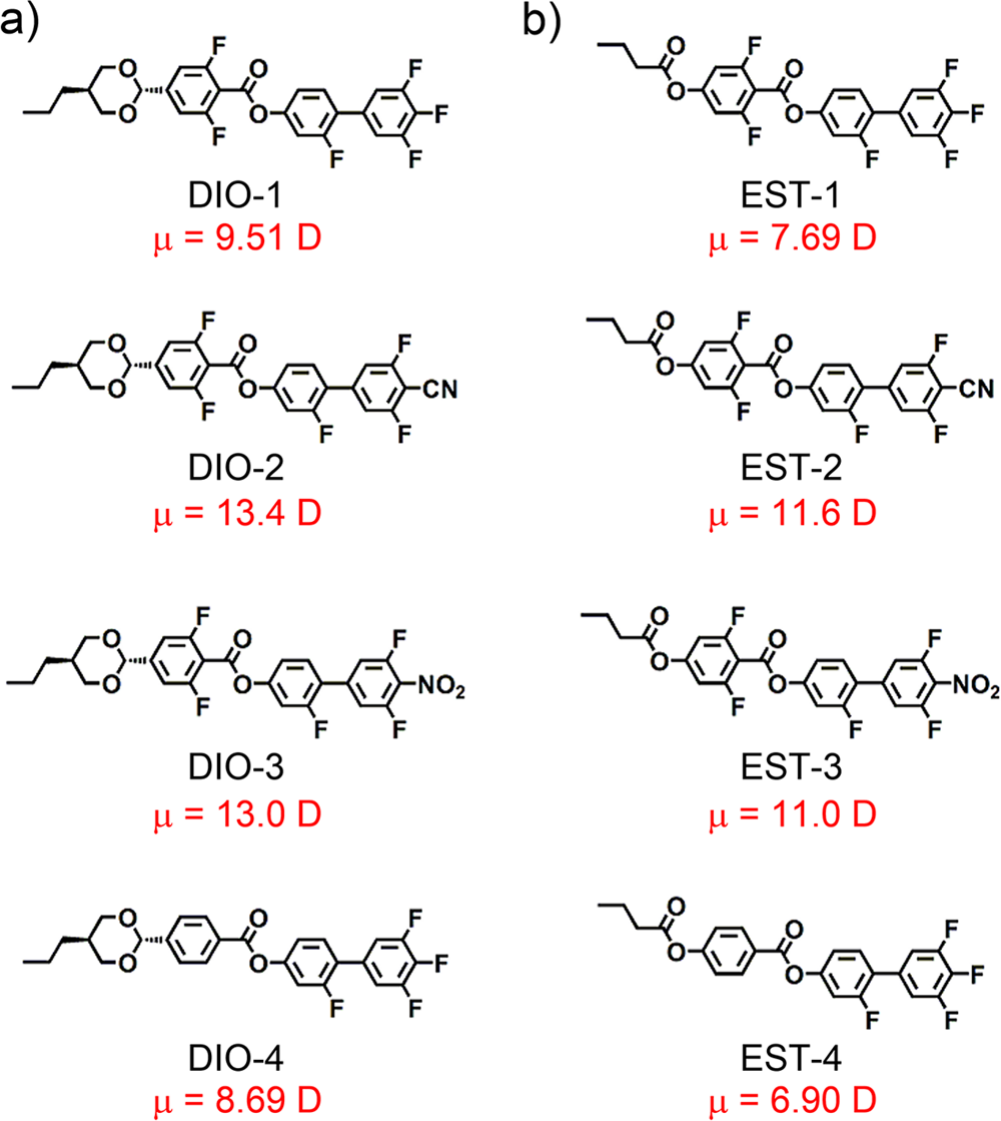
(a) Chemical structures of DIO and its analogs (DIO-n, *n* = 1–4). (b) Chemical structures of DIO analogs consisting of ester linkages instead of 1,3-dioxane structures (EST-*n*, *n* = 1–4). Longitudinal dipole moment values of these analogs were obtained from the optimized structures calculated by the DFT method with the B3LYP/6-31+G(2d,p) basis function^21^ using the Gaussian 16 software package.^22^

To thoroughly elucidate the molecular origin of ferroelectricity, LC analogs with ferroelectric phases must be synthesized, and the relationships between their molecular structures and ferroelectric states must be evaluated. Recently, DIO analogs bearing CN and NO_2_ electron-withdrawing groups (DIO-2 and DIO-3, respectively, Figure 1) were demonstrated to form N_F_ phases.^11,12^ Moreover, an analog consisting of a nonfluorinated benzoate unit (DIO-4) was found to yield a ferroelectric smectic A (SmA_F_) phase, rather than an N_F_ phase.^11^

Although various DIO structural analogs can be designed, their synthesis poses some challenges. Specifically, the formation of the geometric *cis*-isomer during the generation of the 1,3-dioxane unit must be avoided. In addition, alkylated 1,3-propanediol precursors of the 1,3-dioxane unit must be synthesized via a multistep procedure. Overall, these issues result in a decrease in the yield. Furthermore, the 1,3-dioxane unit is thermally unstable, which represents a weakness for practical applications. Conversely, LC molecules containing ester linkages, as exemplified by RM734, prove advantageous for the synthetic extension of the structural variation. Therefore, the conversion of 1,3-dioxane units in the DIO series to ester linkages is anticipated to provide significant advantages for future research endeavors. In this study, newly designed DIO analogs with ester structures instead of 1,3-dioxane structures (EST-*n*, *n* = 1–4) were introduced, and their phase transitions and ferroelectric properties were investigated.

## Structural Design of EST Analogues

Several types of ferroelectric LC molecules incorporate electron-withdrawing groups such as CN and NO_2_ groups. These substituents increase the dipole moments of the entire molecule, thus resulting in the formation of spontaneous polarization that imparts ferroelectric properties. Moreover, DIO analogs bearing CN and NO_2_ groups at their ends (DIO-2 and DIO-3) have been demonstrated to exhibit an N_F_ phase. By contrast, a DIO analog in which the fluorine atoms of the benzene ring adjacent to the 1,3-dioxane structure are replaced by hydrogen atoms (DIO-4) exhibit an SmA_F_ phase instead of an N_F_ phase, despite their dipole moments becoming smaller than that of the original DIO-1. In the EST series, to investigate the impact of the introduction of electron-withdrawing groups and removal of fluorine atoms on the ferroelectricity, four EST analogs were newly designed, as depicted in Figure 1. The synthetic procedures and routes are described in the Supporting Information (Experimental Section). EST analogs were obtained through relatively simple and standard three-to-five-step reactions in higher yields than those observed for the DIO analogs.^11,12^

## Characterization of Phase Transitions and Ferroelectric Properties

**Figure 2:**
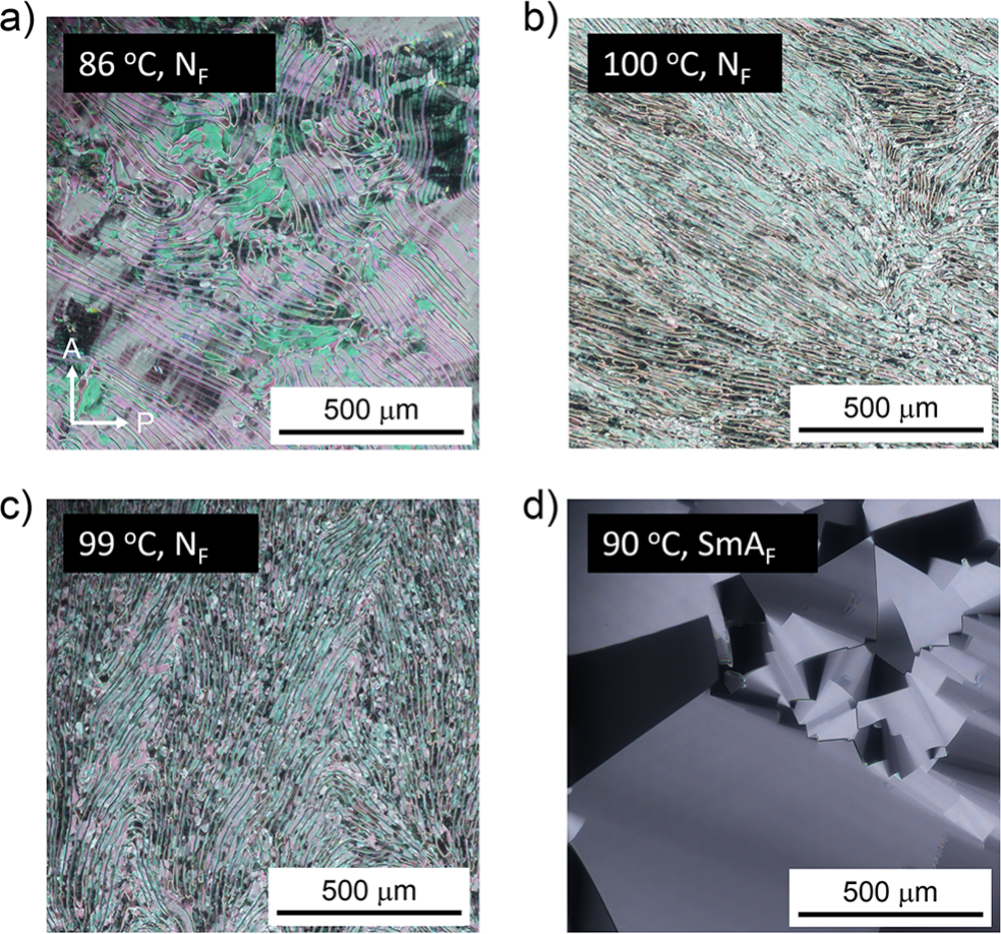
POM images of EST series in their ferroelectric liquid crystal states in the cooling run. (a) EST-1, (b) EST-2, (c) EST-3, (d) EST-4. Samples were injected into cells composed of nontreated glass substrates.

**Figure 3:**
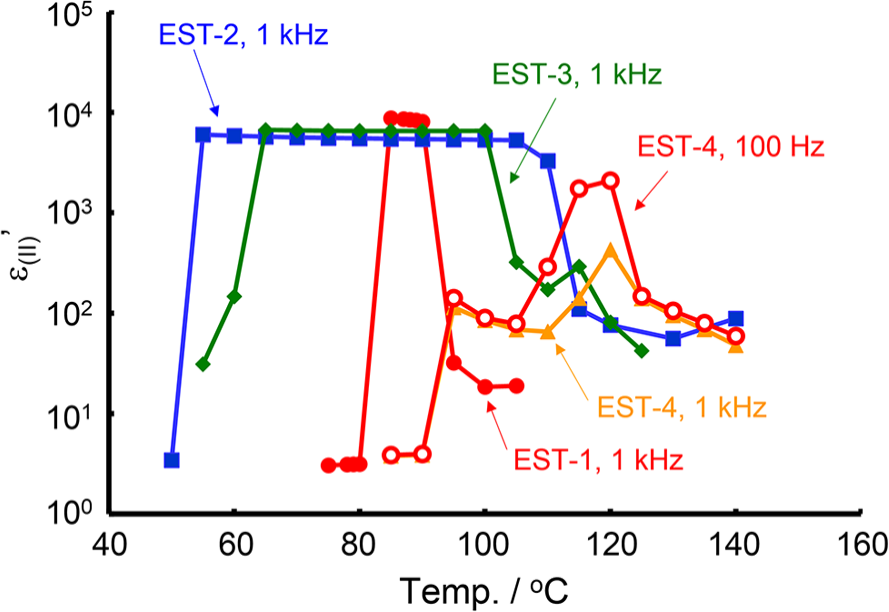
Temperature dependence of the dielectric constant of EST analogs at 100 or 1000 Hz obtained from dielectric relaxation spectra at different temperatures in the cooling run. Closed red circles: EST-1. Closed blue squares: EST-2. Closed green diamonds: EST-3. Closed orange triangles: EST-4 at 1 kHz. Open red circles: EST-4 at 100 Hz. The ε′_(∥)_ is not exactly the same as the dielectric constant along the parallel to the director, generally denoted as ε_∥_′, because for ferroelectric phases, the dielectric constant could be underestimated as ferroelectric phases do not always follow the surface orientation process.

**Figure 4:**
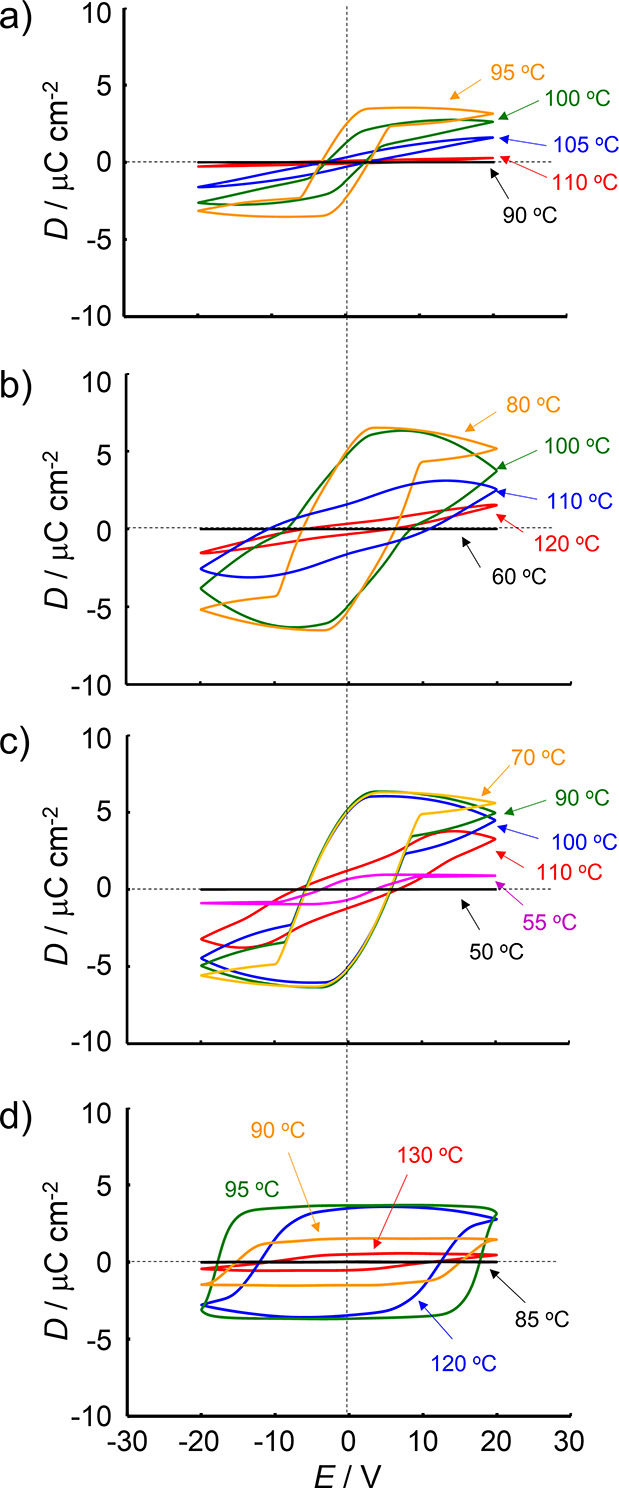
*D*–*E* hysteresis curves obtained from polarization displacement current measurements of EST analogs in homeotropic orientation cells. (a) EST-1 and (b) EST-2 and (c) EST-3 and (d) EST-4. Voltage: 40 V_p–p_. Frequency: 200 Hz for EST-2, 100 Hz for EST-1 and EST-3, and 10 Hz for EST-4.

The phase transitions of the EST analogs were evaluated using differential scanning calorimetry (DSC), polarization optical microscopy (POM), dielectric relaxation spectroscopy, polarization reversal current measurements, and small-angle X-ray scattering measurement (SAXS). The results are summarized in Table 1, and the details of each analysis are shown in Figures 2–4 and Figures S1–S20 in the Supporting Information.

**Table 1:** Phase Transition Behavior of DIO and EST Analogs Obtained through DSC Measurement, POM Observation, Dielectric Relaxation Spectroscopy, and Polarization Reversal Current Measurements^a^

ID	phase transition temperature/°C										
DIO-1	Cr	96			N_F_	69	N_X_	85	N	174	Iso
DIO-2	Cr	121	N_F_′	58	N_F_	65	N_X_	130	N	244	Iso
DIO-3	Cr	98	N_F_′	92	N_F_	96	N_X_	117	N	184	Iso
DIO-4	Cr	129			SmA_F_	146	SmA_F_′	158	N	231	Iso
EST-1	Cr	98			N_F_	89			N	105	Iso
EST-2	Cr	119			N_F_	98	N_X_	127	N	175	Iso
EST-3	Cr	116			N_F_	104	N_X_	112	N	130	Iso
EST-4	Cr	100			SmA_F_	116			N^b^	137	Iso

a The phase transition temperatures of the liquid crystal phases are determined from the DSC results in the second cooling run. The melting points, i.e., the transition temperatures from a crystal (Cr) to another phase, are determined from the DSC charts in the first heating run because unstable crystals are likely to be formed in the second and subsequent runs.

b Cybotactic N phases with smectic short-range ordering.

For EST-1, the DSC chart in the heating run exhibited a sharp exothermic peak at 98 °C and a weak peak at 105 °C (Figure S1). The POM analysis provided a dark field image at 105 °C (Figure S2), clearly indicating the formation of an isotropic (Iso) phase. The DSC chart in the cooling run showed a weak peak at 105 °C, whereas the conventional Schlieren texture characteristic of a nematic (N) phase was noticeable in the POM image at 103 °C (Figure S2). Upon further cooling, a weak peak appeared at 89 °C, whereas a stripe-like texture abruptly appeared at 86 °C (Figure 2a). Upon further cooling, the texture was maintained and then suddenly changed to a texture associated with a crystalline (Cr) state at 77 °C. The dielectric constant remained below 100 from 10 MHz to 10 Hz at 95–105 °C; however, it increased below 10 Hz (Figure S3a). This could be attributed to trace amounts of ionic contaminants. Moreover, the dielectric constant abruptly increased to 10 000 below 10 kHz when the temperature decreased to 85–90 °C (Figure 3 and S3b), thus suggesting the formation of ferroelectric LC phases. The current response to the triangle voltage showed apparent peaks at 95–100 °C (Figure S4), whereas the electric displacement–electric field (*D*–*E*) curves produced apparent hysteresis loops at 95–100 °C (Figure 4a), strongly indicating the formation of ferroelectric LC phases. Based on these results, EST-1 formed a N_F_ phase.

EST-2 and EST-3, which feature electron-withdrawing groups at their terminal para-positions, exhibited large longitudinal dipoles of more than 11.0 D. Because large dipole moments are favorable for the emergence of ferroelectric phases in the DIO series, the same tendency was expected for EST-2 and EST-3. The transition between the LC and Iso phases of EST-2 occurred at approximately 175 °C in both heating/cooling runs (Figure S5). The phase at 150 °C exhibited high fluidity, whereas the POM image at 150 °C does not show characteristic textures such as smectic phases (Figure S6). Thus, EST-2 formed a N phase at this temperature. When the temperature decreased to 100 °C, the texture changed, and a characteristically fine stripe-like texture appeared (Figure 2b), which was similar to that observed for LC molecules showing N_F_ phases. Although only the textural change from a N phase to a N_F_ phase was observed, the DSC profile exhibited an extremely small peak at approximately 127 °C, which could be assigned to the phase transition from a N phase to another N (N_X_) phase. Upon further cooling to 47 °C, the texture remained unchanged but the phase suddenly changed to the Cr phase at 46 °C. The dielectric constant gradually increased as the temperature decreased from 110 °C, eventually reaching over 10 000 in the 100–1000 Hz range (Figure 3 and S7), which further suggests the formation of ferroelectric phases. The polarization reversal current results are shown in Figures 4b and S8. The presence of a partial hysteresis loop was confirmed at 100 °C. In the 80–100 °C range, the *D*–*E* curves showed a large hysteresis loop and the *D* value reached up to 5 μC cm^–2^, which is comparable to that observed for DIO-2 in the N_F_ phase.^12^ At 60 °C, the *D* value significantly decreased, which could be attributed to crystallization. Overall, these results indicated that EST-2 formed an N_F_ phase.

For EST-3, the Iso-LC phase transition occurred at approximately 130 °C (Figure S9). The POM image at 120 °C shows a conventional Schlieren texture, thus indicating the formation of a N phase (Figure S10). Upon cooling, the DSC curve exhibited two weak peaks at 104 and 112 °C, whereas the POM image exhibited a Schlieren texture with a fine sandy-like texture at 110 °C. Upon further cooling, the Schlieren texture completely disappeared, whereas the fine sandy-like texture remained at 105 °C. This sandy-like texture was similar to that observed for the N_F_ phase of DIO-3. On the other hand, a fine stripe-like texture attributed to a N_F_ phase was also obtained for the POM observation of EST-3 injected into a nontreated glass cell (Figure S11). Thus, these results suggest that EST-3 exhibited the N–N_X_–N_F_ phase transition. Accordingly, its dielectric constant reached a value up to approximately 10 000 at 65–100 °C (Figures 3 and S12). Moreover, the current response curves clearly showed peaks and *D*–*E* hysteresis loops at 60–100 °C (Figures 4d and S13). These results strongly indicated the formation of a N_F_ phase.

Next, the phase transition behavior of EST-4 was investigated. The DSC chart showed a peak at 137 °C in both heating and cooling runs (Figure S14), whereas the change in dark-field to bright-field were obtained in the POM observation around this temperature (Figure S15). Evidently, the transition between the N and Iso phases occurred at 137 °C. Upon further cooling, a peak became noticeable in the DSC chart at 116 °C, and a plate-like mosaic texture characteristic of either SmA_F_ or SmX_F_ phases^11^ was observed (Figure 2d). This texture was maintained even when the temperature was decreased. Finally, the texture relative to a Cr state appeared at 84 °C. The dielectric constant increased at 110–120 °C (Figures 3 and S16); specifically, the value was greater than 1000 at 100 Hz. The current response showed strong peaks at 90–120 °C (Figure S17), whereas the *D*–*E* curves featured large hysteresis loops at 90–120 °C (Figure 4d). The maximum *D* value was approximately 3.67 μC cm^–2^, the lowest among the EST series. This result was consistent with the observation that EST-4 possessed the smallest dipole moment. The XRD results highlighted an anisotropic diffraction pattern under an applied magnetic field of approximately 560 mT (Figures S18 and S19). Sharp diffraction spots appeared along the axis parallel to the direction of the magnetic field. In addition, a diffuse scattering of spacings corresponding to the distance between the molecular lateral directions along the axis perpendicular to the direction of the magnetic field was observed. The one-dimensional (1-D) XRD profiles are shown in Figure S20. In particular, at 95–120 °C, strong primary and secondary spots were noticeable at 3.11° and 6.19°, respectively. From these values, the *d* spacing was calculated to be 2.21 nm, which is in good agreement with the molecular length of EST-4 (2.17 nm). These results clearly demonstrate that EST-4 formed an SmA_F_ phase. Furthermore, 1-D XRD profiles at 125–140 °C exhibited weak diffractions associated with Sm layers, and a mosaic-like texture characteristic of the Sm phase was partially observed in POM images at 130 and 135 °C. These results suggest that this phase is a cybotactic N phase with smectic short-range ordering.

## Comparison of the Effect of Structural Variations on the Phase Transition Behavior and Ferroelectricity

Finally, the impact of replacing the 1,3-dioxane structure with an ester linkage was explored. As presented in Table 1, the Iso–N transition temperatures of the EST analogs were lower than those of the corresponding DIO analogs. In particular, the transition temperature of EST-4 was approximately 100 °C lower compared with that of DIO-4. DIO analogs can form additional phases such as the N_X_, N_F_′, and SmA_F_′ phases, whereas the phase transitions in EST analogs occur more straightforwardly. Particularly, EST-1 and EST-4 exhibited only a transition from the N phase to the ferroelectric phases. Compared with the DIO analogs, the phase transition temperatures of the N_F_ and SmA_F_ phases of the EST analogs are more favorable. DIO-3 exhibited the N_F_ phase in a narrow temperature range, which immediately changed to the N_F_′ and Cr phases. The N_F_ phase of EST-3 was stable over a wide temperature range, which is advantageous for practical applications. Although the detailed mechanism of the substitution effect on the ester structure is not fully understood, we assume that the ester structure reduces the strong intermolecular interactions based on the 1,3-dioxane structure to simplify the phase transition and reduce the phase transition temperatures. As reported in our previous study, only DIO analogs with dipole moments greater than 8.5 D can form ferroelectric phases. Surprisingly, EST-4 afforded the SmA_F_ phase despite its small dipole moment of 6.90 D. Li et al. demonstrated through machine learning experiments that dipole moments larger than 9 are desirable for ferroelectricity.^10^ Our findings contradict the conventional notion that LC molecules with large dipole moments are favorable for the emergence of ferroelectric LC phases. To achieve a ferroelectric state with dipole–dipole interactions between molecules, the dipole moment must be sufficiently large to allow their interactions to withstand thermal fluctuations. In this case, the following equation must hold for a simple calculation.^23^
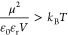
where μ, ε_0_, ε_r_, *V*, *k*_B_, and *T* represent the dipole moment, dielectric constant of vacuum, relative dielectric constant of substance, volume, Boltzmann constant, and absolute temperature, respectively. To approximately calculate the critical dipole moment, the volume (0.54 nm^3^) estimated from the expected density^9^ (∼1.3 g cm^–3^) and ferroelectric–paraelectric phase transition temperature of EST-4 (389 K) were substituted into the equation. The relative dielectric constant used here should be the value when the dipole moment is completely disordered; however, the value measured in this experiment cannot be used because of strong dipole–dipole interactions. Based on the measured dielectric constants in nematic LC materials with dipole moments close to that of EST-4 and Kirkwood correlation factors that are not excessively large,^24^ the relative dielectric constant 22–27 was used in this calculation. The lowest dipole moment that satisfied the above equation was approximately 7.1–7.9 D. A dipole moment of 6.9 D for EST-4 was found to be below the minimum threshold at which the dipole interaction could withstand the thermal fluctuations. Therefore, other favorable interactions may possibly be influencing EST-4 and contributing to its ferroelectric properties, in addition to dipole–dipole interactions.

In this study, EST analogs consisting of ester in place of 1,3-dioxane structures were synthesized, and their phase transitions and ferroelectric properties were characterized. Accordingly, all EST analogs exhibited ferroelectric LC phases, and the temperatures at which these phases emerged tended to decrease and expand. Moreover, the EST analogs showed ferroelectric smectic phases despite their smaller dipole moments. These features are beneficial for practical applications. Notably, the EST analogs exhibited ferroelectricity despite a dipole moment lower than 7.0 D. The dipole moment of 6.90 D in the case of compound EST-4 represents the smallest value reported thus far among ferroelectric LCs with spontaneous polarization in the director direction. Because EST analogs lack a 1,3-dioxane structure, the separation of cis/trans isomers was not required, and synthetic modifications were readily performed, thus yielding various structural analogs. Our results provide valuable insights for the design of ferroelectric LCs.

## Supporting Information Available

Detailed synthetic procedures; the results of DSC, POM, dielectric relaxation spectroscopy, polarization reversal current measurements, and SAXS measurements (PDF)

## References

[ref1] Bisoyi H.; Li Q. Liquid Crystals: Versatile Self-organized Smart Soft Materials. Chem. Rev. 2022, 122, 4887–4926. 10.1021/acs.chemrev.1c00761. 34941251

[ref2] Horiuchi S.; Tokura Y. Organic Ferroelectrics. Nat. Mater. 2008, 7, 357–366. 10.1038/nmat2137. 18432209

[ref3] Lagerwall J.; Scalia G. A new Era for Liquid Crystal Research: Applications of Liquid Crystals in Soft Matter Nano-, Bio- and Microtechnology. Curr. Appl. Phys. 2012, 12, 1387–1412. 10.1016/j.cap.2012.03.019.

[ref4] Lee J.; Lee S.-D. Ferroelectric Liquid Crystalline Ordering of Rigid Rods with Dipolar Interactions. Mol. Cryst. Liq. Cryst. 1994, 254, 395–403. 10.1080/10587259408036088.

[ref5] Nishikawa H.; Shiroshita K.; Higuchi H.; Okumura Y.; Haseba Y.; Yamamoto S.; Sago K.; Kikuchi H. A Fluid Liquid-Crystal Material with Highly Polar Order. Adv. Mater. 2017, 29, 1702354 10.1002/adma.201702354. 29023971

[ref6] Chen X.; Korblova E.; Dong D.; Wei X.; Shao R.; Radzihovsky L.; Glaser M. A.; Maclennan J. E.; Bedrov D.; Walba D. M.; Clark N. A. First-Principles Experimental Demonstration of Ferroelectricity in A Thermotropic Nematic Liquid Crystal: Polar Domains and Striking Electro-Optics. Proc. Nat. Acad. Sci. 2020, 117, 14021–14631. 10.1073/pnas.2002290117. 32522878 PMC7322023

[ref7] Mandle R.; Cowling S. J.; Goodby J. W. A Nematic to Nematic Transformation Exhibited by A Rod-Like Liquid Crystal. Phys. Chem. Chem. Phys. 2017, 19, 11429–11435. 10.1039/C7CP00456G. 28422219

[ref8] Manabe A.; Bremer M.; Kraska M. Ferroelectric Nematic Phase at and below Room Temperature. Liq. Cryst. 2021, 48, 1079 10.1080/02678292.2021.1921867.

[ref9] Mandle R. J.; Sebastian N.; Martinez-Perdiguero J.; Mertelj A. On the Molecular Origins of the Ferroelectric Splay Nematic Phase. Nat. Commun. 2021, 12, 4962 10.1038/s41467-021-25231-0. 34400645 PMC8367997

[ref10] Li J.; Nishikawa H.; Kougo J.; Zhou J.; Dai S.; Tang W.; Zhao X.; Hisai Y.; Huang M.; Aya S. Development of Ferroelectric Nematic Fluids with Giant-ε Dielectricity and Nonlinear Optical Properties. Sci. Adv. 2021, 7, eabf5047 10.1126/sciadv.abf5047. 33883139 PMC8059932

[ref11] Kikuchi H.; Matsukizono H.; Iwamatsu K.; Endo S.; Anan S.; Okumura Y. Fluid Layered Ferroelectrics with Global C_∞v_ Symmetry. Adv. Sci. 2022, 9, 2202048 10.1002/advs.202202048. PMC9475520 35869031

[ref12] Matsukizono H.; Iwamatsu K.; Endo S.; Okumura Y.; Anan S.; Kikuchi H. Synthesis of Liquid Crystals Bearing 1,3-Dioxane Structures and Characterization of Their Ferroelectricity in the Nematic Phase. J. Mater. Chem. C 2023, 11, 6183–6190. 10.1039/D2TC05363B.

[ref13] Nishikawa H.; Sano K.; Araoka F. Anisotropic Fluid with Phototunable Dielectric Permittivity. Nat. Commun. 2022, 13, 1142 10.1038/s41467-022-28763-1. 35241651 PMC8894468

[ref14] Nishikawa H.; Kuwayama M.; Nihonyanagi A.; Dhara B.; Araoka F. Rapid, Solvent-minimized and Sustainable Access to Various Types of Ferroelectric-fluid Molecules by Harnessing Mechano-chemical Technology. J. Mater. Chem. C 2023, 11, 12525–12542. 10.1039/D3TC02212A.

[ref15] Thoen J.; Cordoyiannis G.; Jiang W.; Mehl G. H.; Glorieux C. Phase transitions Study of the Liquid Crystal DIO with a Ferroelectric Nematic, a Nematic, and an Intermediate Phase and of Mixtures with the Ferroelectric Nematic Compound RM734 by Adiabatic Scanning Calorimetry. Phys. Rev. 2023, 107, 014701 10.1103/PhysRevE.107.014701. 36797863

[ref16] Yadav N.; Panarin Y.; Vij J.; Jiang W.; Mehl G. Two Mechanisms for the Formation of the Ferronematic Phase Studies by Dielectric Spectroscopy. J. Mol. Liq. 2023, 378, 121570 10.1016/j.molliq.2023.121570.

[ref17] Chen X.; Martinez V.; Korblova E.; Freychet G.; Zhernenkov M.; Glaser M.; Wang C.; Zhu C.; Radzihovsky L.; Maclennan J.; Walba D.; Clark N. The Smectic ZA Phase: Antiferroelectric Smectic Order as a Prelude to the Ferroelectric Nematic. Prog. Nat. Acad. Sci. 2023, 120, e2217150120 10.1073/pnas.2217150120. PMC9974471 36791101

[ref18] Sebastian N.; Lovsin M.; Berteloot B.; Osterman N.; Petelin A.; Mandle R.; Aya S.; Huang M.; Drevenšek-Olenik I.; Neyts K.; Mertelj A. Polarization Patterning in Ferroelectric Nematic Liquids via Flexoelectric Coupling. Nat. Commun. 2023, 14, 3029 10.1038/s41467-023-38749-2. 37230977 PMC10213025

[ref19] Matoba Y.; Uemura S.; Funahashi M. Diastereomeric Effect on Bulk Photovolatic Property and Polarized Electroluminescence in Ferroelectric Liquid Crystals Containing an Extended π-Conjugated Unit. Bull. Chem. Soc. Jpn. 2023, 96, 247–256. 10.1246/bcsj.20230011.

[ref20] Takahashi H.; Kohri M.; Kishikawa K. Axially Polar-Ferroelectric Columnar Liquid Crystalline System That Maintains Polarization upon Switching to the Crystalline Phase: Implications for Maintaining Long-Term Polarization Information. ACS Appl. Nano Mater. 2023, 6, 10531–10538. 10.1021/acsanm.3c01508.

[ref21] Ju S.-P.; Huang S.-C.; Lin K.-H.; Chen H.-Y.; Shen T.-K. Prediction of Optical and Dielectric Properties of 4-Cyano-4-pentylbiphenyl Liquid Crystals by Molecular Dynamics Simulation, Coarse-Grained Dynamics Simulation, and Density Functional Theory Calculation. J. Phys. Chem. C 2016, 120, 14277–14288. 10.1021/acs.jpcc.5b12222.

[ref22] Frisch M. J.; Trucks G. W.; Schlegel H. B.; Scuseria G. E.; Robb M. A.; Cheeseman J. R.; Scalmani G.; Barone V.; Petersson G. A.; Nakatsuji H.; Li X.; Caricato M.; Marenich A. V.; Bloino J.; Janesko B. G.; Gomperts R.; Mennucci B.; Hratchian H. P.; Ortiz J. V.; Izmaylov A. F.; Sonnenberg J. L.; Williams-Young D.; Ding F.; Lipparini F.; Egidi F.; Goings J.; Peng B.; Petrone A.; Henderson T.; Ranasinghe D.; Zakrzewski V. G.; Gao J.; Rega N.; Zheng G.; Liang W.; Hada M.; Ehara M.; Toyota K.; Fukuda R.; Hasegawa J.; Ishida M.; Nakajima T.; Honda Y.; Kitao O.; Nakai H.; Vreven T.; Throssell K.; Montgomery J. A. Jr.; Peralta J. E.; Ogliaro F.; Bearpark M. J.; Heyd J. J.; Brothers E. N.; Kudin K. N.; Staroverov V. N.; Keith T. A.; Kobayashi R.; Normand J.; Raghavachari K.; Rendell A. P.; Burant J. C.; Iyengar S. S.; Tomasi J.; Cossi M.; Millam J. M.; Klene M.; Adamo C.; Cammi R.; Ochterski J. W.; Martin R. L.; Morokuma K.; Farkas O.; Foresman J. B.; Fox D. J. Gaussian 16, Revision C.01; Gaussian, Inc.: Wallingford, CT, 2016.

[ref23] Lavrentovich O. Ferroelectric Nematic Liquid Crystal, A Century in Waiting. Prog. Nat. Acad. Sci. 2020, 117, 14629–14631. 10.1073/pnas.2008947117. PMC7334533 32541021

[ref24] McDonnell D.; Raynes E.; Smith R. Dipole Moments and Dielectric Properties of Fluorine Substituted Nematic Liquid Crystals. Liq. Cryst. 1989, 6, 515–523. 10.1080/02678298908034171.

