# Supplementary material for: Exploring the Impact of Linkage Structure in Ferroelectric Nematic and Smectic Liquid Crystals
Source: J Phys Chem Lett. 2024 Apr 10;15(15):4212–7. doi: 10.1021/acs.jpclett.3c03492 (PMC11033931; doi:10.1021/acs.jpclett.3c03492)
Supplement: Supplementary file 1 — jz3c03492_si_001.pdf [file jz3c03492_si_001.pdf]

Supporting Information for:

Exploring the Impact of Linkage Structure in Ferroelectric Nematic and Smectic Liquid Crystals

Hiroyuki Matsukizono,[a] Yusuke Sakamoto,[b] Yasushi Okumura,[a] and Hirotsugu Kikuchi,[a*]

[a] Kyushu University, Institute for Materials Chemistry and Engineering, 6-1 Kasuga-Koen, Kasuga, Fukuoka 816-8580, Japan.

[b] Kyushu University, Interdisciplinary Graduate School of Engineering Sciences, 6-1 Kasuga-Koen, Kasuga, Fukuoka 816-8580, Japan.

Reagents

Potassium carbonate (K_2CO_3), sodium chlorite ($NaClO_2$), sodium dihydrogen phosphate (NaH_2PO_4), anhydrous sodium sulfate (Na_2SO_4), triethylamine (NEt_3) and 1-ethyl-3-(3-dimethylaminopropyl)carbodiimide hydrochloride (WSC) were obtained from Wako Pure Chemical Co., Ltd. Benzyl bromide, *n*-butyl lithium hexane solution (1.6 mol L^{-1}), 4-dimethylaminopyridine (DMAP), 10% palladium carbon (wetted with ca. 55% water) (Pd/C), 3,5-difluorophenol and other fluorinated benzene derivatives were purchased from Tokyo Chemical Industry Co., Ltd. other reagents containing acryl chloride were obtained from commercial sources. Fluorinated phenylphenol compounds, 3-fluoro-4-(3,4,5-trifluorophenyl)phenol, 4-(4-cyano-3,5-difluorophenyl)-3-fluorophenol and 4-(3,5-difluoro-4-nitro-phenyl)-3-fluorophenol were synthesized according to literature procedure[1] or obtained from JNC Co., Ltd. These reagents and solvents were used without any purification.

Measurements

Measurements for synthesis of EST series: NMR spectra were recorded on a JNM-ECZ400 spectrometer (JEOL Co., Ltd.) at 400 MHz for ^{1}H NMR. Chemical shifts in ^{1}H NMR spectra are given in parts per million (ppm) downfield using tetramethylsilane as an internal standard. The high-resolution mass spectroscopy (HRMS) was conducted on a JMS-700 instrument (JEOL Co., Ltd.) with a fast atom bombardment (FAB) mode.

Differential scanning calorimetry (DSC) measurements: DSC profiles were recorded on a DSC 1 STARe System calorimeter (Mettler Toledo, Switzerland) with a dedicated aluminum pan at a scanning rate of 5 °C min^{-1}.

Polarized optical microscopy (POM) observations: POM observation was conducted using Nikon ECLIPSE LV100NPOL with a DS-Ri2 camera. For the measurement, three types of cells were prepared: no orientation treatment, planar orientation (parallel to the substrate but with free in-plane orientation), and homeotropic orientation (perpendicular to the substrate). The planar orientation cells were prepared by spin coating method using a toluene solution of poly(methyl methacrylate) (PMMA) on glass substrates and successive drying at 120 °C for 2 h. Homeotropic orientation on a glass substrate was performed by vapor deposition method using octadecyltrimethoxysilane (180 °C, 2 h, 1 bar).

Dielectric relaxation spectroscopy: Dielectric relaxation spectra were measured at 1–10 MHz using an impedance/gain phase analyzer (SI 1260, Solatron Metrology) at an applied voltage of 0.1 V_{rms}. The samples were injected in the homeotropically oriented cells with an electrode area of 1 cm^2 and a cell thickness of 10 μm. The indium thin oxide (ITO) electrode resistance and capacitance were measured using the empty cell. the obtained values were used for the correction of the sample impedance to afford the sample dielectric

constants.

Polarization reversal current measurements: a waveform generator (2411B, Toyo Technica), an analog-to-digital converter (WaveBook 516A, Toyo Technica), and a current–voltage/charge–voltage (I–V/Q–V) converter (Model 6254C, Toyo Technica) were constructed. Samples were injected into a homeotropically oriented ITO cell with an electrode area if 1 cm^2 and a cell thickness of 10 μm. The polarization reversal current was measured using the triangular wave method at an applied voltage of ±20 V and a frequency of 10–200 Hz. The electron displacement-electron field (*D-E*) hysteresis loops were obtained by integrating the measured currents.

Small angle X-ray scattering (SAXS) measurements: SAXS experiments were performed at Kyushu University Beamline (SAGA-LS/BL06). The samples were poured into a hole of the stage (diameter = 3 mm, depth = 1 mm) equipped with a pair of magnets (magnetic field = approximately 560 mT). The stage was set at which the direction of magnetic field (the direction of the director of EST series) aligned perpendicular to the direction of X-ray irradiation (detector distance = 179 mm, wavelength = 0.12 nm). The measurement was performed in the cooling run at 145–90 °C with an aging time of 1 min and an exposure time of 10 s. One-dimensional (1-D) profiles (2θ versus intensity plots) were obtained by the integration of the obtained SAXS images.

Quantum chemical calculation: The most stable structures and dipole moment (μ) of EST series were obtained using DFT calculation with the B3LYP/6-31+G(2d,p) basis function[2] in the Gaussian16 software program.[3]

1. Synthesis

1-1. Synthesis of 3-fluoro-4-(3,4,5-trifluoropheny)phenyl 4-butanoyloxy-2,6-difluorobenzoate (**EST-1**)

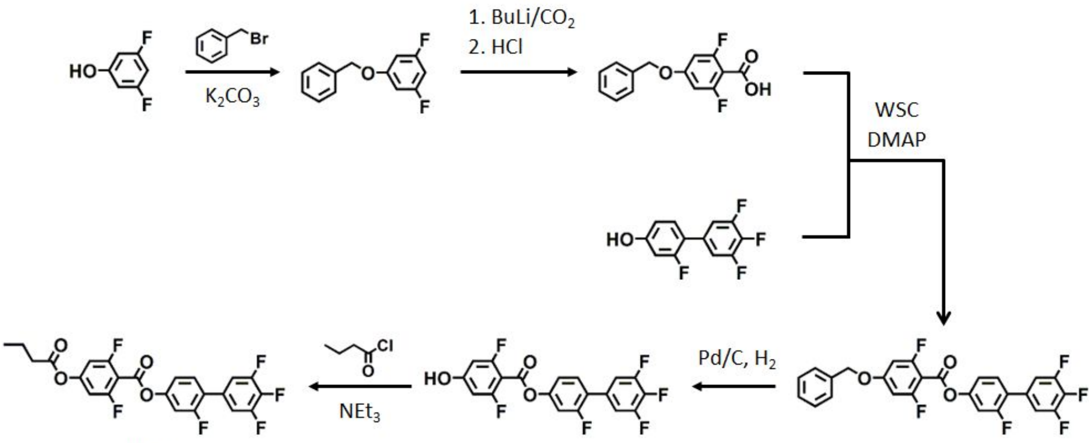

EST-1

1-1-1. Synthesis of benzyl 3,5-difluorophenyl ether

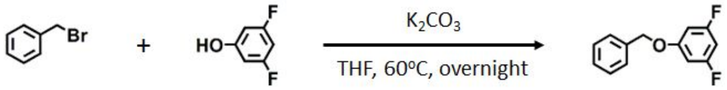

Benzyl bromide 6.91 g (40.4 mmol) and 3,5-difluorophenol 5.74 g (44.1 mmol) were dissolved in dehydrated THF (44 mL). To the solution, K_2CO_3 9.20 g (66.6 mmol) were added and then the mixture was stirred at 60°C overnight. After cooling, insoluble solids were filtrated and the filtrates were evaporated. The residual yellow liquid was dissolved in n-hexane (100 mL) and washed three times with distilled water (100 mL). The organic later was dried over anhydrous Na_2SO_4 and then evaporated. The residues were purified by column chromatography (eluent: n-hexane) to give colorless liquids. Yield: 8.35 g (94.8 %). ^{1}H NMR (400 MHz, $CDCl_3$, ppm): δ 7.40–7.29 (m, 5H; phenyl), 6.53-6.43 (m, 2H, phenyl), 6.42 (tt, 1H, J = 10, 2.1 Hz, phenyl), 5.02 (s, 2H, CH_2).

1-1-2. Synthesis of 4-benzyloxy-2,6-difluorobenzoic acid

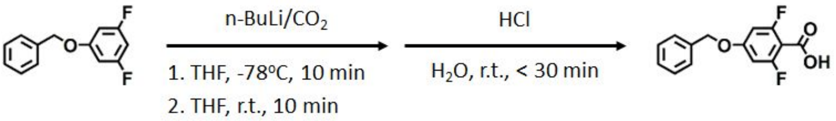

To a THF solution (50 mL) of benzyl 3,5-difluorophenyl ether 7.00 g (31.8 mmol) was added dropwise 1.6 M *n*-butyl lithium n-hexane solution (25 mL, 40 mmol, 1.3 equiv.) at −78°C under N_2 atmosphere. After stirring

for 10 min, dry ice blocks 28.0 g (640 mol) was added and then stirred at −78°C for 10 min. The mixture was stirred at ambient temperature for 30 min, H_2O (ca. 5 mL) was added and the solvents were removed by rotary evaporator. The residues were dissolved in distilled water (50 mL) and then its pH was adjusted to 2–3 by an addition of 1 M HCl aq. The precipitates were collected by suction filtration and washed with distilled water. After drying under reduced pressure, white solids were obtained. Yield: 5.29 g (62.9%). ^{1}H NMR (400 MHz, DMSO-d_6, ppm): δ 7.46–7.34 (m, 5H; phenyl), 6.91 (dd, *J* = 14, 5.2 Hz, 2H, phenyl), 5.19 (s, 2H, CH_2).

1-1-3. Synthesis of 3-fluoro-4-(3,4,5-trifluoropheny)phenyl 4-benzyloxy-2,6-difluorobenzoate

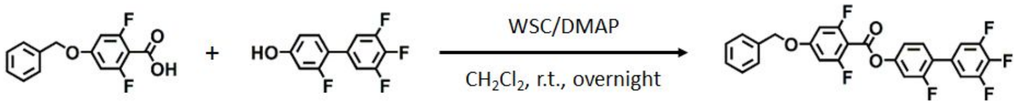

4-benzyloxy-2,6-difluorobenzoic acid 5.28 g (20.0 mmol), DMAP 244 mg (2.00 mmol) and 3-fluoro-4-(3,4,5-trifluorophenyl)phenol 5.33 g (22.0 mmol) were dissolved in CH_2Cl_2 (100 mL). To the solution, WSC 4.39 g (22.9 mmol) was added and then the mixture was stirred at rt. overnight. The resulting precipitates were collected by suction filtration and washed with CH_2Cl_2. The precipitates were further obtained from the reprecipitation from filtrates with MeOH. There precipitates were combined and then dried in vacuo to afford white solids. Yield: 7.44 g (76.2%). ^{1}H NMR (400 MHz, $CDCl_3$, ppm): δ 7.45–7.37 (m, 6H, phenyl), 7.20–7.12 (m, 4H, phenyl), 6.63 (d, *J* = 10 Hz, 2H), 5.12 (s, 2H, CH_2).

1-1-4. Synthesis of 3-fluoro-4-(3,4,5-trifluoropheny)phenyl 2,6-difluoro-4-hydroxybenzoate

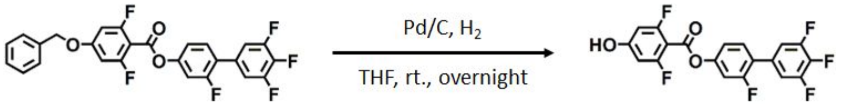

3-fluoro-4-(3,4,5-trifluoropheny)phenyl 4-benzyloxy-2,6-difluorobenzoate 7.33 g (15.0 mmol) was dissolved in THF (60 mL). To the solution, 5 wt% Pd/C 367 mg was added and then the mixture was stirred at ambient temperature overnight under H_2 atmosphere. After black solids was removed by suction filtration, the filtrates were evaporated. The residues were purified by reprecipitation from acetone/n-hexane to obtain white solids. Yield: 5.57 g (93.3%). ^{1}H NMR (400 MHz, DMSO-d_6, ppm): δ11.5 (br, 1H, COOH), 7.68 (t, 1H, *J* = 8.6 Hz, phenyl), 7.61 (t, 2H, *J* = 7.8 Hz, phenyl), 7.42 (dd, 1H, *J* = 11, 2.2 Hz, phenyl), 7.25 (dd, 1H, *J* = 8.8, 2.2 Hz, phenyl), 6.65 (d, 2H, *J* = 11 Hz, phenyl).

1-1-5. Synthesis of **EST-1**

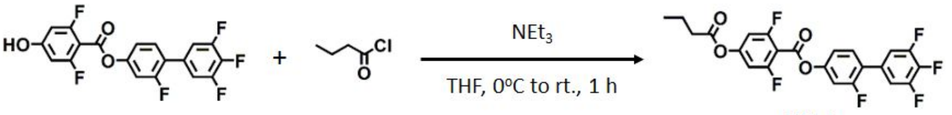

EST-1

3-fluoro-4-(3,4,5-trifluoropheny)phenyl 2,6-difluoro-4-hydroxybenzoate 796 mg (2.00 mmol) and NEt_3 222 mg (2.19 mmol) were dissolved in dehydrated THF (10 mL) and the solution was cooled to 0 °C in an ice bath. To the solution, butyryl chloride 234 mg (2.20 mmol) dissolved in THF (5 mL) was added dropwise and the mixture was stirred at ambient temperature for 1 day. After the precipitates were removed by suction filtration, the filtrates were concentrated and the residue was dissolved in CH_2Cl_2. The solution was washed three times with ultra-pure water and then dried over anhydrous Na_2SO_4. After evaporation, the solids were purified by silica-gel column chromatography (eluent; n-hexane/CH_2Cl_2 = 1:1 by Vol.) followed by recrystallization from n-hexane/CH_2Cl_2 to give white solids. Yield: 85.8%. ^{1}H NMR (400 MHz, $CDCl_3$, ppm): δ 7.46-7.42 (m, 1H, phenyl), 7.20–7.15 (m, 4H, phenyl), 6.90 (d, 2H, *J* = 8.8 Hz, phenyl), 2.58 (t, 2H, *J* = 7.2 Hz, CH_2-C=O), 1.80 (sext, 2H, *J* = 7.4 Hz, CH_3-C$\underline{H_2}$-), 1.06 (t, 3H, *J* = 7.2 Hz, CH_3). HRMS (FAB+) m/z: calcd for $C_{23}H_{14}F_6O_4$: 468.0796; found: 469.0875 (M+H).

1-2. Synthesis of 4-(4-cyano-3,5-difluoropheny)-3-fluoro-phenyl 4-butanoyloxy-2,6-difluorobenzoate (**EST-2**)

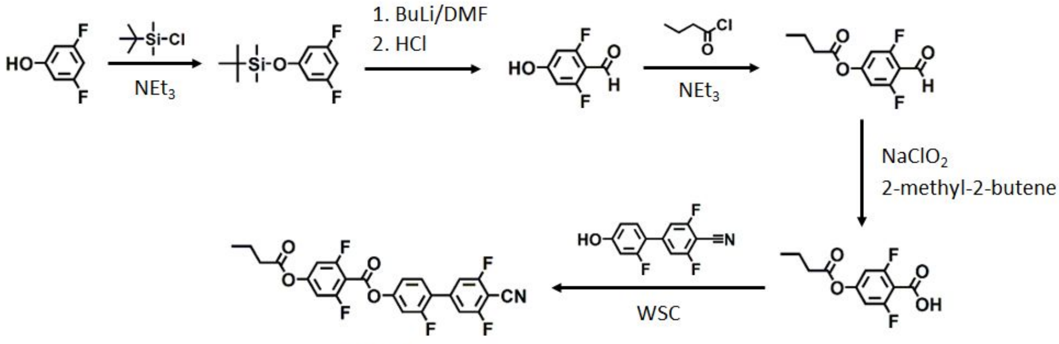

EST-2

1-2-1. Synthesis of *tert*-butyldimethylsilyl 3,5-difluorophenyl ether

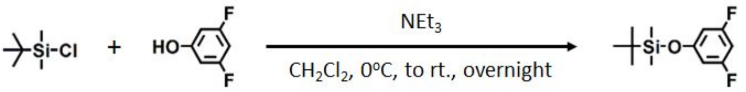

To a CH_2Cl_2 solution (20 mL) of 3,5-difluorophenol 2.86 g (22.0 mmol) and NEt_3 2.20 g (21.7 mmol) was added

dropwise *tert*-butyldimethylsilyl chloride 3.00 g (19.9 mmol) at 0 °C. The mixture was stirred at ambient temperature for 2 days and then precipitates were removed by suction filtration. The filtrates were washed three times with distilled water and then dried over anhydrous Na_2SO_4. After evaporation, the residue was dried under reduced pressure to give pale yellow liquid. Yield: 4.87 g (>99%). ^{1}H NMR (400 MHz, $CDCl_3$, ppm): δ 6.45–6.40 (m, 1H, phenyl), 6.39-6.33 (m, 2H, phenyl), 0.98 (s, 9H, *tert*-butyl), 0.22 (s, 6H, CH_3-Si).

1-2-2. Synthesis of 2,6-difluoro-4-hydroxybenzaldehyde

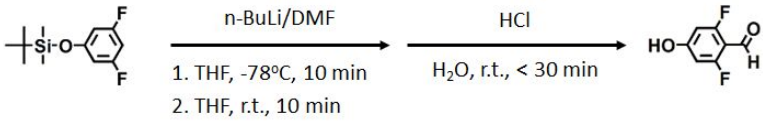

tert-butyldimethylsilyl 3,5-difluorophenyl ether 9.54 g (39.0 mmol) was dissolved in dehydrated THF (30 mL) and then the solution was cooled to −78 °C in a CO_2/acetone bath. To the solution was slowly added *n*-BuLi hexane solution (ca. 1.6 M, 30 mL, 48 mmol) and the solution was stirred at the temperature for 10 min. After that, dehydrated DMF 3.14 g (43.0 mmol) was added. After the solution was stirred at −78 °C for 10 min and then stirred at ambient temperature. After quenching by H_2O, THF was removed by evaporation and then the residues were dissolved in H_2O. The pH of the solution was adjusted to ca. 4 by an addition of 1 M HCl aq. and then the resulting precipitates were collected by suction filtration. After the solids were washed by CH_2Cl_2, white solids were obtained. Yield: 4.21 g (68.2%). ^{1}H NMR (400 MHz, CD_3OD, ppm): δ 10.1 (s, 1H, CHO), 6.45 (d, 2H, J = 11 Hz, phenyl).

1-2-3. Synthesis of 4-butanoyloxy-2,6-difluorobenzaldehyde

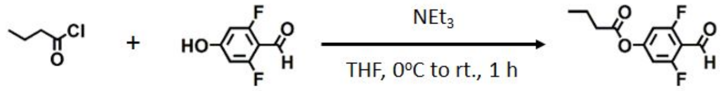

To a CH_2Cl_2 solution (20 mL) containing 2,6-difluoro-4-hydroxybenzaldehyde 1.02 g (6.45 mmol) and NEt_3 715 mg (7.07 mmol) was added dropwise a CH_2Cl_2 solution (3 mL) of n-butanoyl chloride 750 mg (7.04 mmol) at 0 °C. After the mixture was stirred at ambient temperature for 2 days, the mixture was added to CH_2Cl_2 and then washed with $NaHCO_3$ aq. followed by distilled water. The organic layer was dried over anhydrous Na_2SO_4 and then concentrated. The residue was purified by silica–gel column chromatography (eluent: hexane/CH_2Cl_2 = 1:1 by Vol.) to give colorless liquid. Yield: 1.43 g (97.3%). ^{1}H NMR (400 MHz, $CDCl_3$, ppm): δ10.3 (s, 1H, CHO), 6.85 (d, 2H, J = 9.6 Hz, phenyl), 2.57 (t, 2H, J = 7.4 Hz, -CH_2-COO-), 1.83-1.74 (m, 2H, CH_3-

$C\underline{H}_2$-), 1.04 (t, 3H, *J* = 7.4 Hz, CH_3-).

1-2-4. Synthesis of 4-butanoyloxy-2,6-difluorobenzoic acid

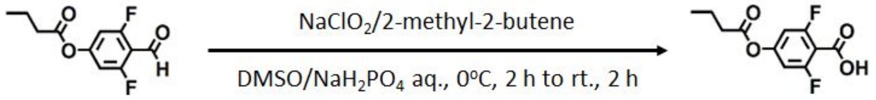

To a DMSO solution (2.0 mL) of 4-butanoyloxy-2,6-difluorobenzaldehyde 1.34 g (5.87 mmol) were added 2-methyl-2-butene 2.10 g (29.9 mmol) and an aqueous solution (5.0 mL) of NaH_2PO_4 2.88 g (24.0 mmol). To the mixture, an aqueous solution (4.0 mL) of $NaClO_2$ 2.17 g (24.0 mmol) was added dropwise at 0 °C and then the mixture was stirred at 0 °C for 2 h. After stirring at ambient temperature for 2 h, the pH of the mixture was adjusted to ca. 3 by an addition of 1 M HCl aq. The resulting precipitates were collected by suction filtration and then dried under reduced pressure. White solids were obtained. Yield: 1.01 g (70.1%). ^{1}H NMR (400 MHz, $CDCl_3$, ppm): δ6.84 (d, 2H, *J* = 8.8 Hz, phenyl), 2.56 (t, 2H, *J* = 7.2 Hz, -CH_2-COO-), 1.83–1.74 (m, 2H, CH_3-$C\underline{H}_2$-), 1.05 (t, 3H, *J* = 7.4 Hz, CH_3-).

1-2-5. Synthesis of **EST-2**

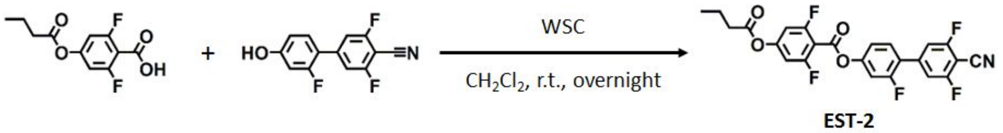

To CH_2Cl_2 (30 mL) were added 4-butanoyloxy-2,6-difluorobenzoic acid 977 mg (4.00 mmol) and 4-(4-cyano-3,5-difluorophenyl)-3-fluorophenol 1.20 g (4.82 mmol). To the mixture, WSC 1.05 g (5.48 mmol) was slowly added and the mixture was stirred at ambient temperature for 2.5 h. The mixture was added to CH_2Cl_2 (50 mL) and then washed three times with ultra-pure water (50 mL). After the organic layer was dried over anhydrous Na_2SO_4, solvents were evaporated. The residue was purified by silica-gel column chromatography (eluent: n-hexane/CH_2Cl_2 = 1:2 by Vol.) followed by recrystallization from hexane/CH_2Cl_2 to afford white crystals. Yield: 638 mg (33.6%). ^{1}H NMR (400 MHz, $CDCl_3$, ppm): δ7.52–7.48 (m, 1H, phenyl), 7.28–7.21 (m, 5H, phenyl), 6.91 (d, 2H, *J* = 9.2 Hz, phenyl), 2.59 (t, 2H, *J* = 7.4 Hz, -CH_2-COO-), 1.84-1.75 (m, 2H, CH_3-$C\underline{H}_2$-), 1.06 (t, 3H, *J* = 7.4 Hz, CH_3-). HRMS (FAB+) m/z: calcd for $C_{24}H_{14}F_5NO_4$: 475.0843; found: 476.0923 (M+H).

1-3. Synthesis of 4-(3,5-difluoro-4-nitropheny)-3-fluorophenyl 4-butanoyloxy-2,6-difluorobenzoate (**EST-3**)

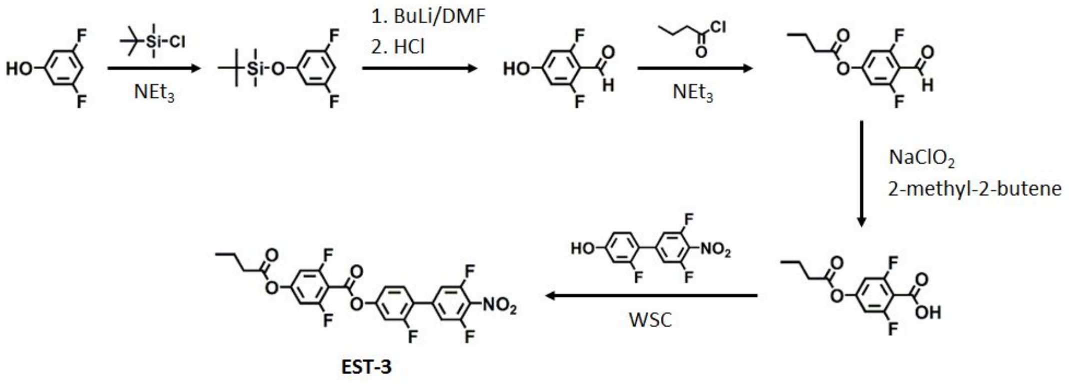

To CH_2Cl_2 (10 mL) were added 4-butanoyloxy-2,6-difluorobenzoic acid 900 mg (3.69 mmol) and 4-(4-nitro-3,5-difluorophenyl)-3-fluorophenol 1.15 g (4.27 mmol). To the mixture, WSC 860 mg (4.49 mmol) was slowly added and the mixture was stirred at ambient temperature for 2 h. The mixture was added to CH_2Cl_2 (100 mL) and then washed three times with ultra-pure water (100 mL). After the organic layer was dried over anhydrous Na_2SO_4, solvents were evaporated. The residue was purified by silica-gel column chromatography (eluent: n-hexane/CH_2Cl_2 = 1:1 by Vol.) followed by recrystallization from n-hexane/CH_2Cl_2 to afford a white solid. Yield: 724 mg (40.0%). ^{1}H NMR (400 MHz, $CDCl_3$, ppm): δ7.84–7.79 (m, 3H, phenyl), 7.55 (dd, 1H, J = 12, 2.0 Hz, phenyl), 7.38–7.34 (m, 3H, phenyl), 2.62 (t, 2H, J = 7.0 Hz, -CH_2-COO-), 1.72–1.63 (m, 2H, CH_3-C$\underline{H}_2$-), 0.99 (t, 3H, J = 7.6 Hz, CH_3-). HRMS (FAB+) m/z: calcd for $C_{23}H_{14}F_5NO_6$: 495.0741; found: 496.0821 (M+H).

1-4. Synthesis of 3-fluoro-4-(3,4,5-trifluoropheny)phenyl 4-butanoyloxybenzoate (**EST-4**)

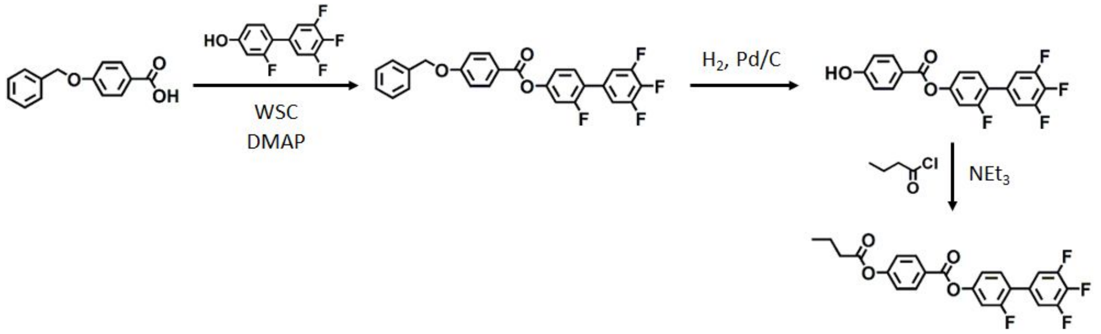

1-4-1. Synthesis of 3-fluoro-4-(3,4,5-trifluorophenyl)phenyl 4-benzyloxybenzoate

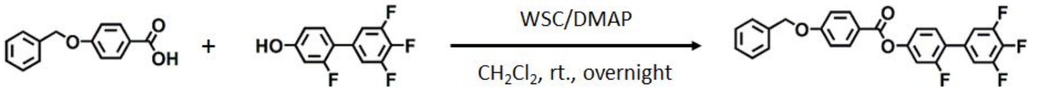

In a 200 mL round-bottom flask, 4-benzyloxybenzoic acid 4.56 g (20.0 mmol) and 3-fluoro-4-(3,4,5-trifluorophenyl)phenol 5.33 g (22.0 mmol), DMAP 2.44 g (20.0 mmol) were dissolved in CH_2Cl_2 (100 mL). To the solution, WSC 4.50 g (23.5 mmol) was added and then the mixture was stirred at ambient temperature overnight. The resulting precipitate was collected by suction filtration and washed with CH_2Cl_2 to give a desired product. The filtrate was washed with HCl aq. followed by distilled water. After drying over anhydrous Na_2SO_4, the solvents were evaporated. The residue was washed with MeOH to afford the desired product as white solid. Yield: 8.69 g (96.0%). ^{1}H NMR (400 MHz, DMSO-d_6, ppm): δ8.10 (d, *J* = 8.8 Hz, phenyl), 7.70–7.60 (m, 3H, phenyl), 7.50–7.22 (m, 12H, phenyl), 5.26 (s, 2H, -CH_2-O-).

1-4-2. Synthesis of 3-fluoro-4-(3,4,5-trifluorophenyl)phenyl 4-hydroxybenzoate

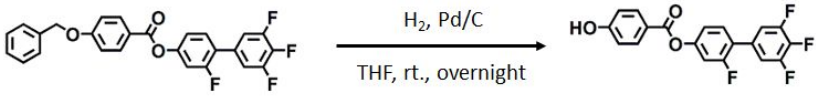

To a THF solution (100 mL) of 3-fluoro-4-(3,4,5-trifluorophenyl)phenyl 4-benzyloxybenzoate 8.69 g (19.2 mmol) was added 5 wt% Pd/C 900 mg (10 wt%) and then the mixture was stirred in H_2 atmosphere at ambient temperature for 2 days. After solids were filtrated off, the filtrate was concentrated. The residue was purified by reprecipitation from acetone/hexane to afford a white solid. Yield: 6.33 g (90.9%). ^{1}H NMR (400 MHz, DMSO-d_6, ppm): δ10.6 (s, 1H, OH), 8.01 (d, *J* = 8.4 Hz, phenyl), 7.69–7.59 (m, 3H, phenyl), 7.43 (dd, 1H, *J* =

11.8, 2.2 Hz, phenyl), 7.26 (dd, 1H, *J* = 8.8, 2.4 Hz, phenyl), 6.95 (d, 2H, *J* = 9.2 Hz, phenyl).

1-4-3. Synthesis of **EST-4**

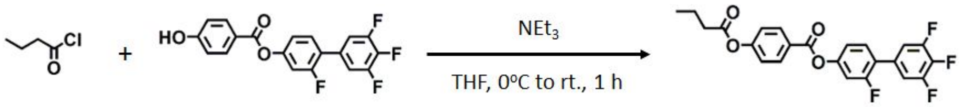

EST-4

To a THF solution (30 mL) of 3-fluoro-4-(3,4,5-trifluorophenyl)phenyl 4-hydroxybenzoate 1.09 g (3.00 mmol) and NEt_3 334 mg (3.30 mmol) was added dropwise butyryl chloride 330 mg (3.10 mmol) at 0 °C. The mixture was stirred at ambient temperature for 1 h and then the resulting precipitate was filtered off. The filtrate (ca. 50 mL) was washed with distilled water (50 mL) followed by ultra-pure water (50 mL × 2). After drying over anhydrous Na_2SO_4, the solvents were removed by rotary evaporator. The crude product 1.53 g was purified by silica-gel column chromatography (hexane/CH_2Cl_2 volume ratio of 1:1 to 1:2) followed by recrystallization from n-hexane/CH_2Cl_2. White solids were obtained. Yield: 1.15 g (85.8%). ^{1}H NMR (400 MHz, $CDCl_3$, ppm): δ8.24 (d, *J* = 8.8 Hz, 2H, phenyl), 7.46–7.41 (m, 1H, phenyl), 7.28–7.26 (m, 2H, phenyl), 7.19 (t, 2H, *J* = 7.2 Hz, phenyl), 7.14–7.10 (m, 2H, phenyl), 2.60 (t, 2H, *J* = 7.2 Hz, -CH_2-C=O), 1.86–1.77 (m, 2H, CH_3-C$\underline{H}_2$-), 1.07 (t, 3H, *J* = 7.4 Hz, CH_3-). HRMS (FAB+) m/z: calcd for $C_{23}H_{16}F_4O_4$: 432.0985; found: 433.1064 (M+H).

2. Characterization of EST-1

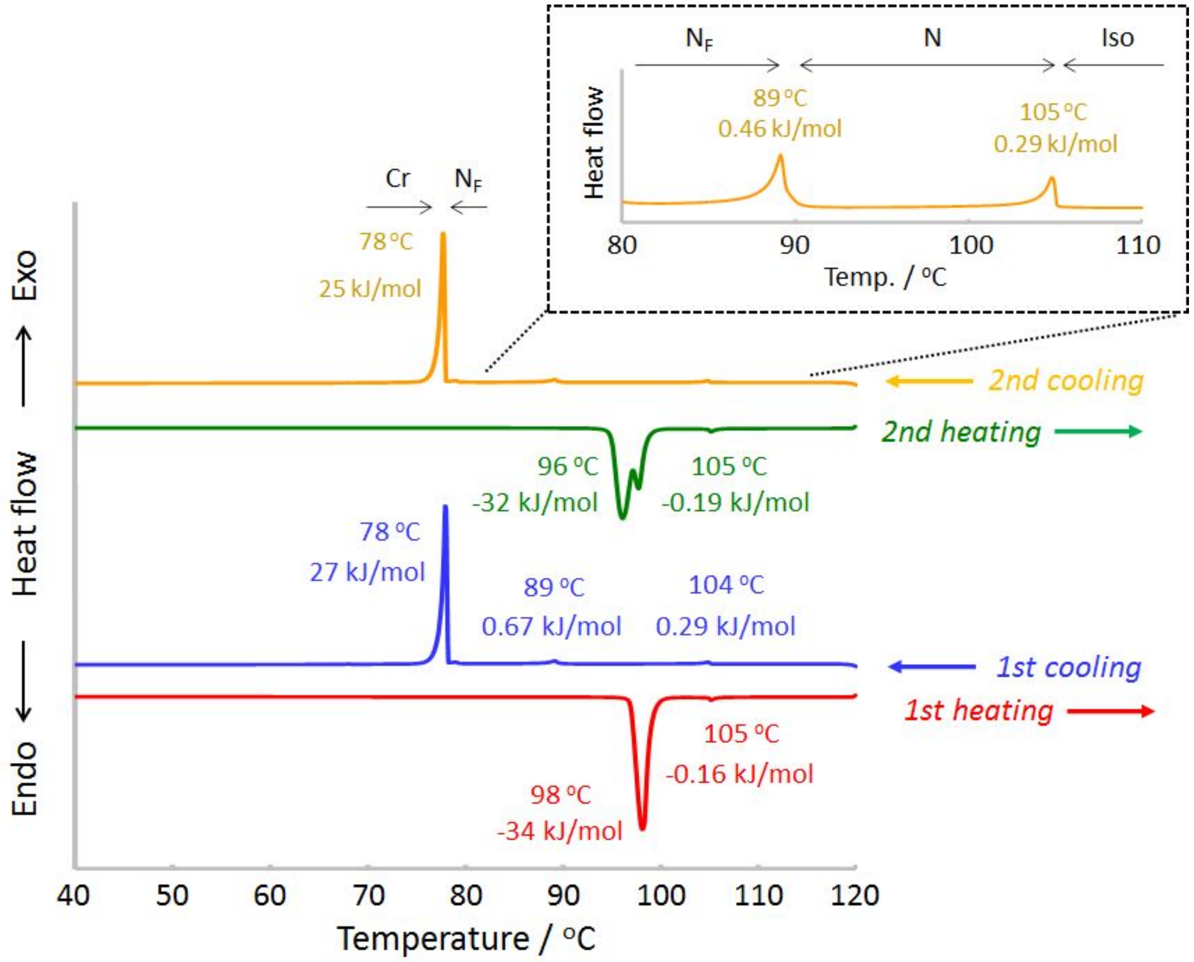

Figure S1 DSC charts of **EST-1**. Rate: 5 °C min^{-1}. The magnified profile with the identification of phase transitions in the 2nd cooling run is shown.

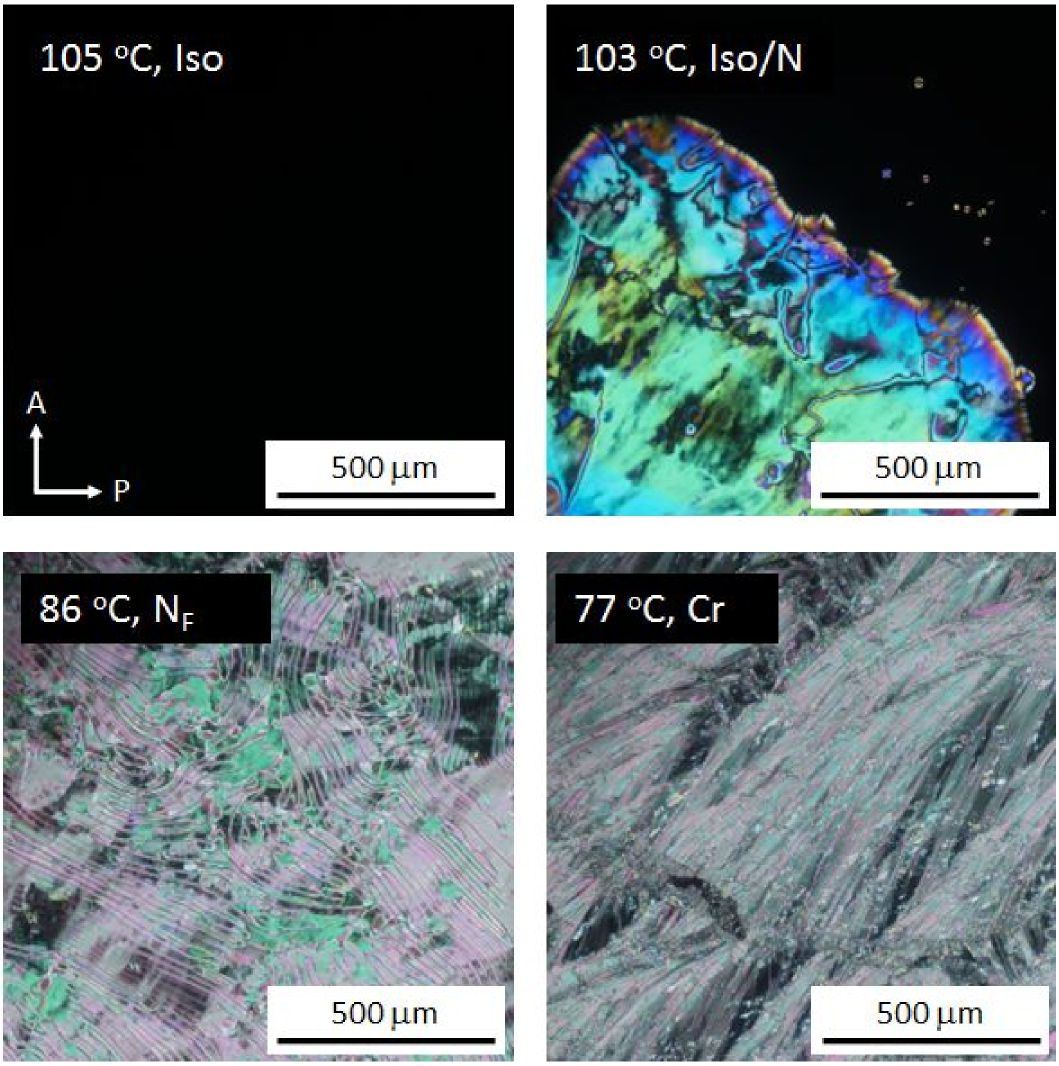

Figure S2 POM images of **EST-1** in the cooling run. **EST-1** was injected in the cell composed of non-treated glass substrates and then POM observation was performed.

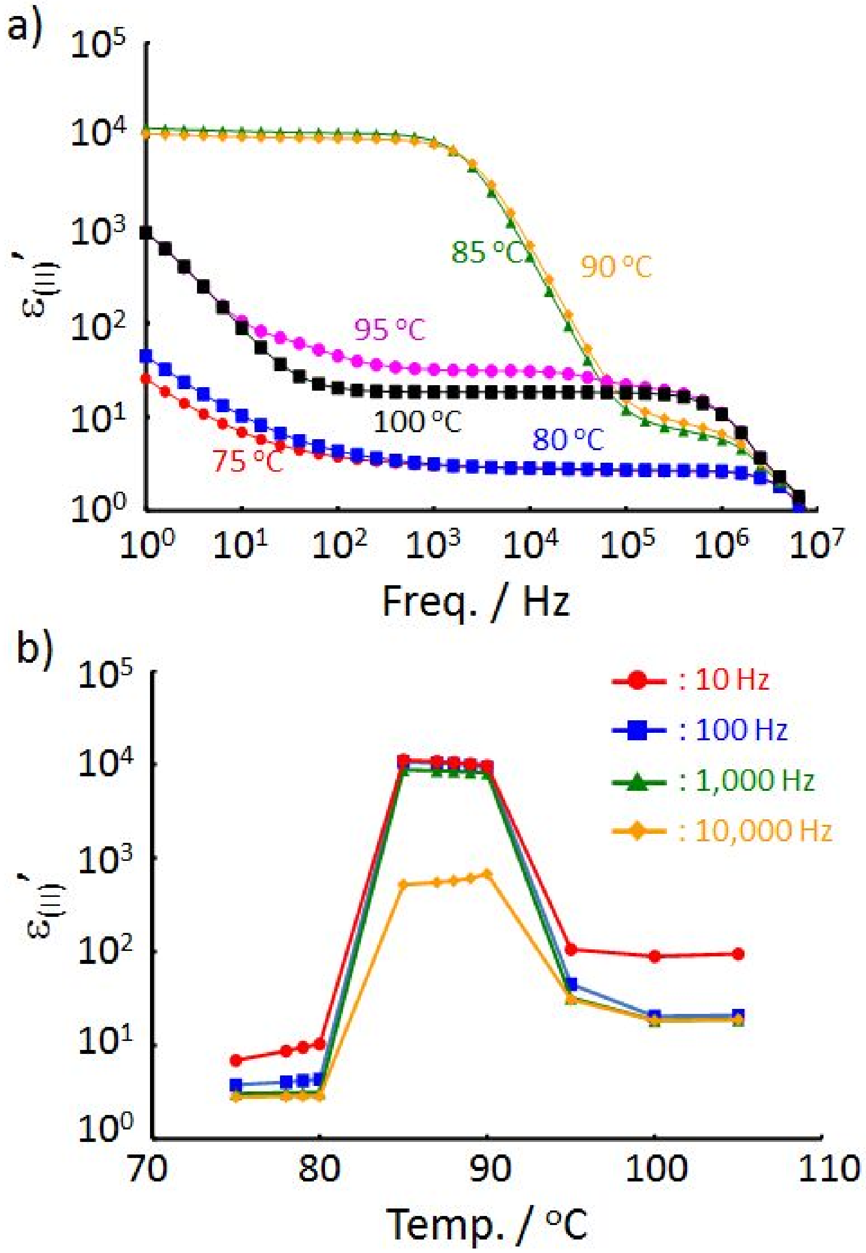

Figure S3 a) Frequency dependences of dielectric constant of **EST-1** in the 1st cooling run. b) Temperature dependences of dielectric constant of **EST-1** at different frequencies. **EST-1** was inserted in the cell consisting in homeotropically oriented glass substrates and then the measurements were carried out.

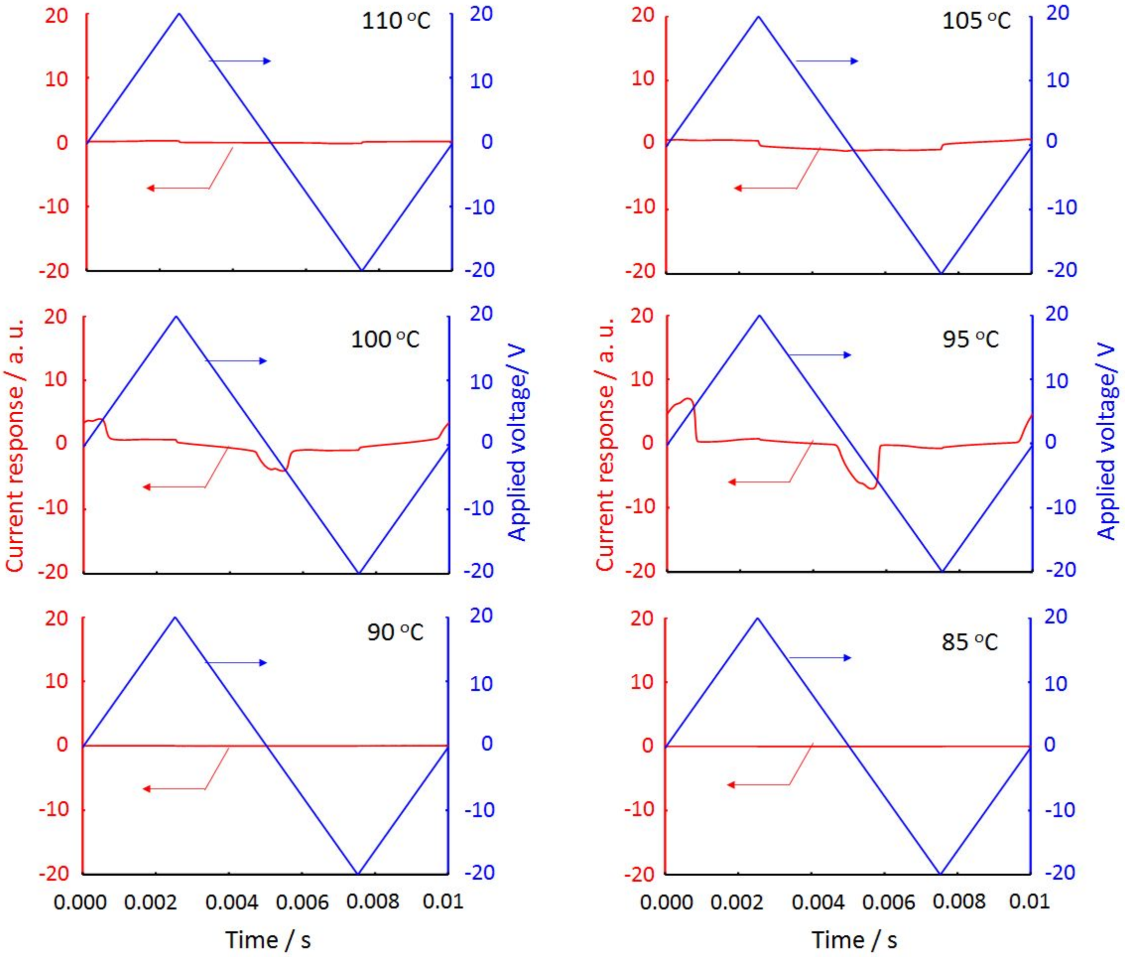

Figure S4 Switching current response of **EST-1** under an applied triangular wave voltage in the cooling run. $V_{P\text{–}P}$ = 40 V. *f* = 200 Hz. Red and black lines denote current response and applied triangular wave voltage, respectively.

3. Characterization of EST-2

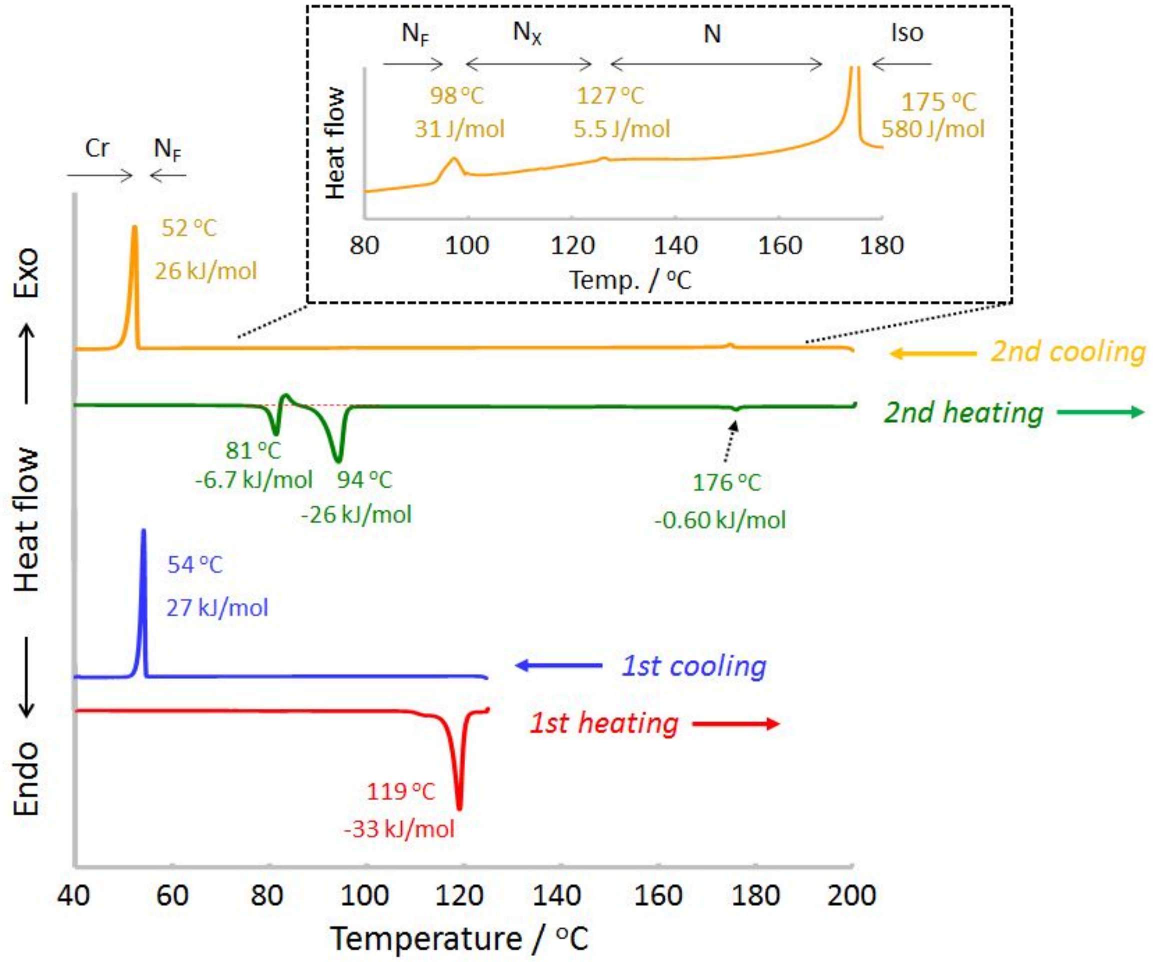

Figure S5 DSC charts of **EST-2**. Rate: 5 °C min^{-1}. The magnified profile with the identification of phase transitions in the 2nd cooling run is shown.

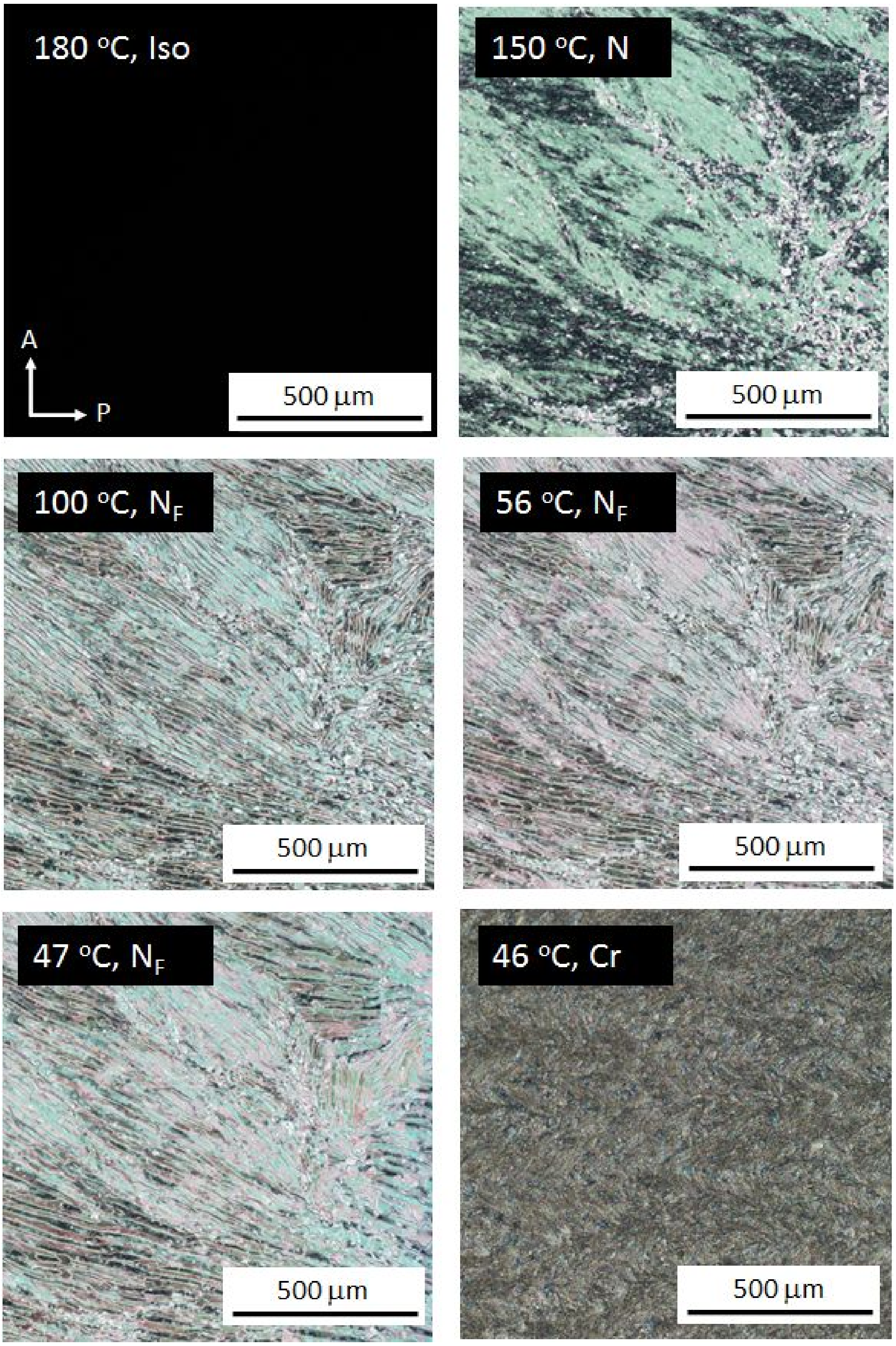

Figure S6 POM images of **EST-2** in the cooling run. **EST-2** was introduced in the cell composed of non-treated glass substrates and then POM observation was performed.

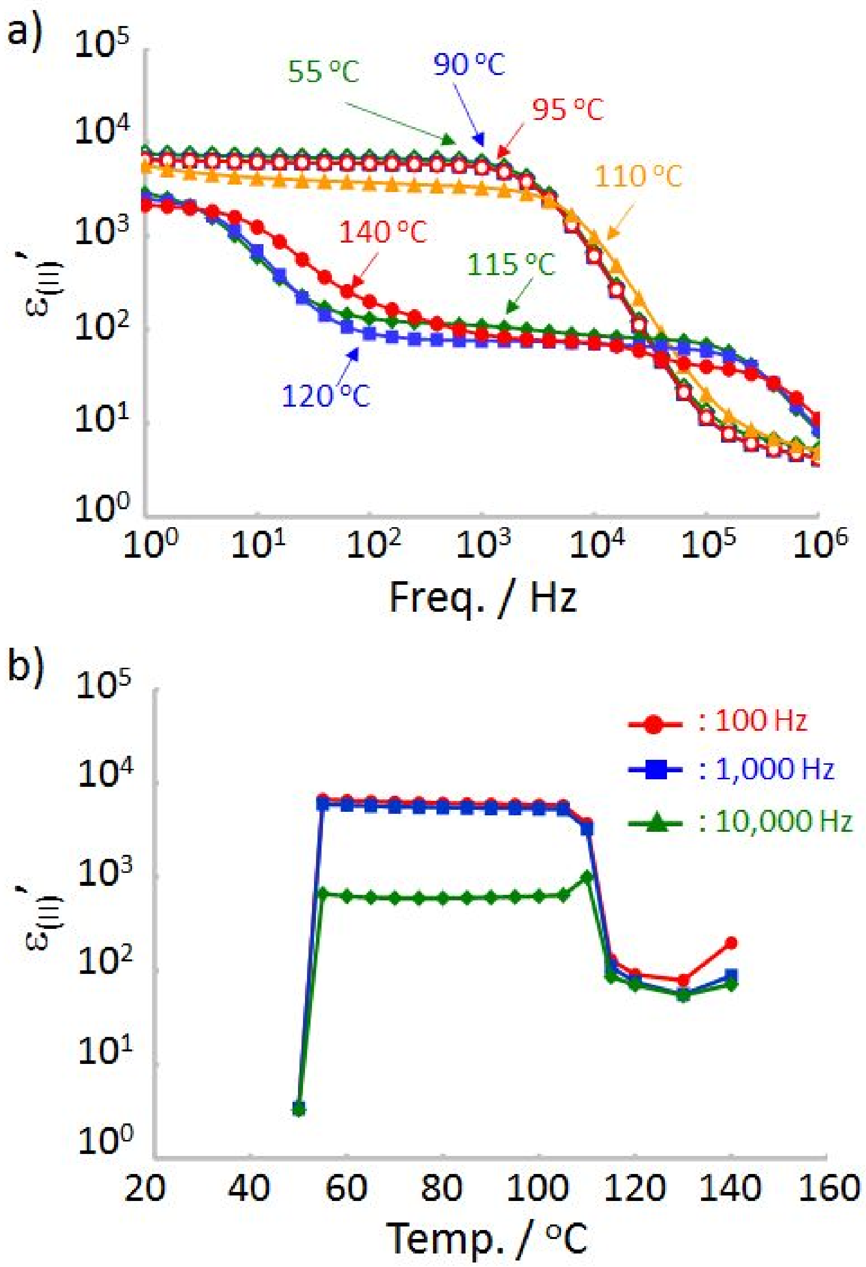

Figure S7 a) Frequency dependences of dielectric constant of **EST-2** in the 1st cooling run. b) Temperature dependences of dielectric constant at different frequencies. **EST-2** was inserted in the cell consisting in homeotropically oriented glass substrates and then the measurements were carried out.

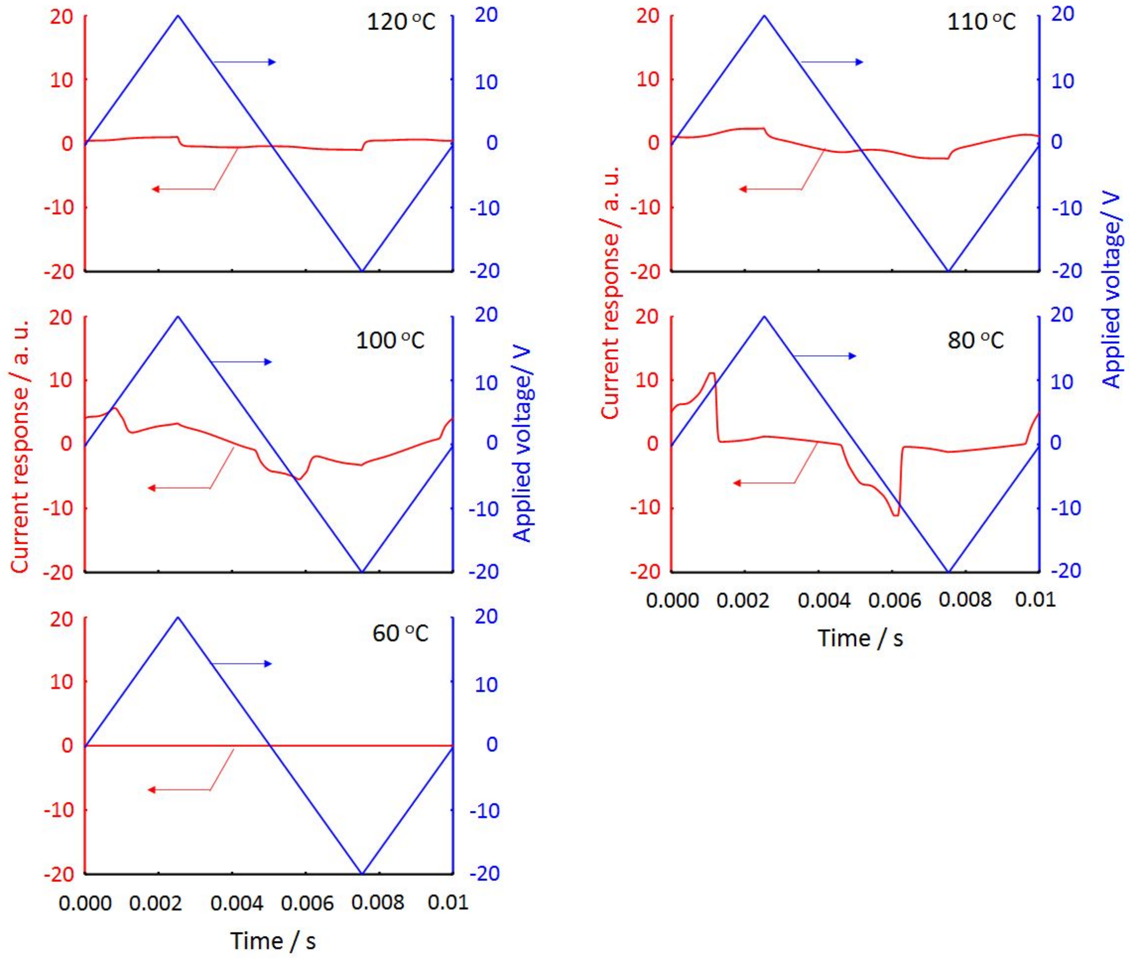

Figure S8 Switching current response of **EST-2** under an applied triangular wave voltage in the cooling run. $V_{P\text{–}P}$ = 40 V. f = 200 Hz. Red and black lines denote current response and applied triangular wave voltage, respectively.

4. Characterization of EST-3

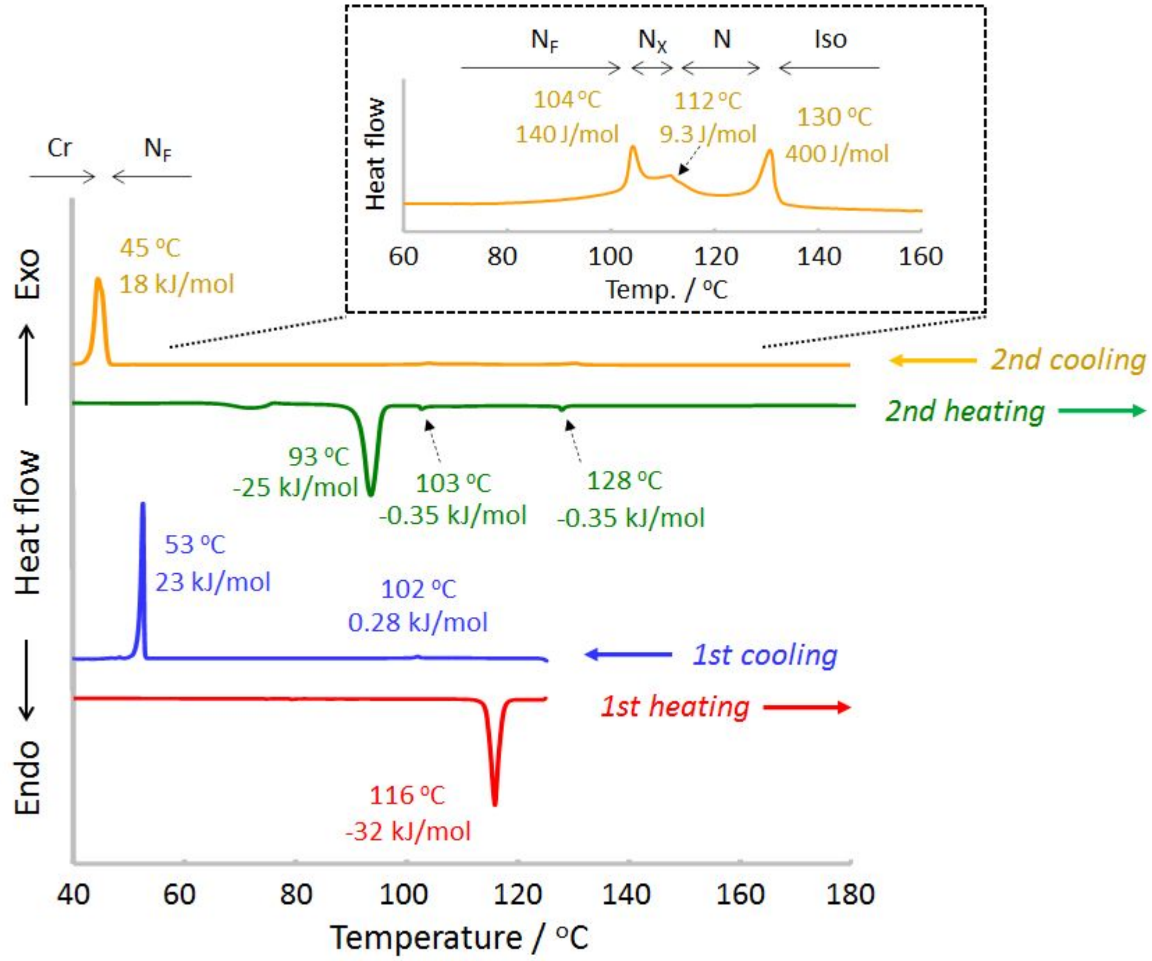

Figure S9 DSC charts of **EST-3**. Rate: 5°C min^{-1}. The identification of phase transitions in the 2nd cooling run is shown. The magnified profile with the identification of phase transitions in the 2nd cooling run is shown.

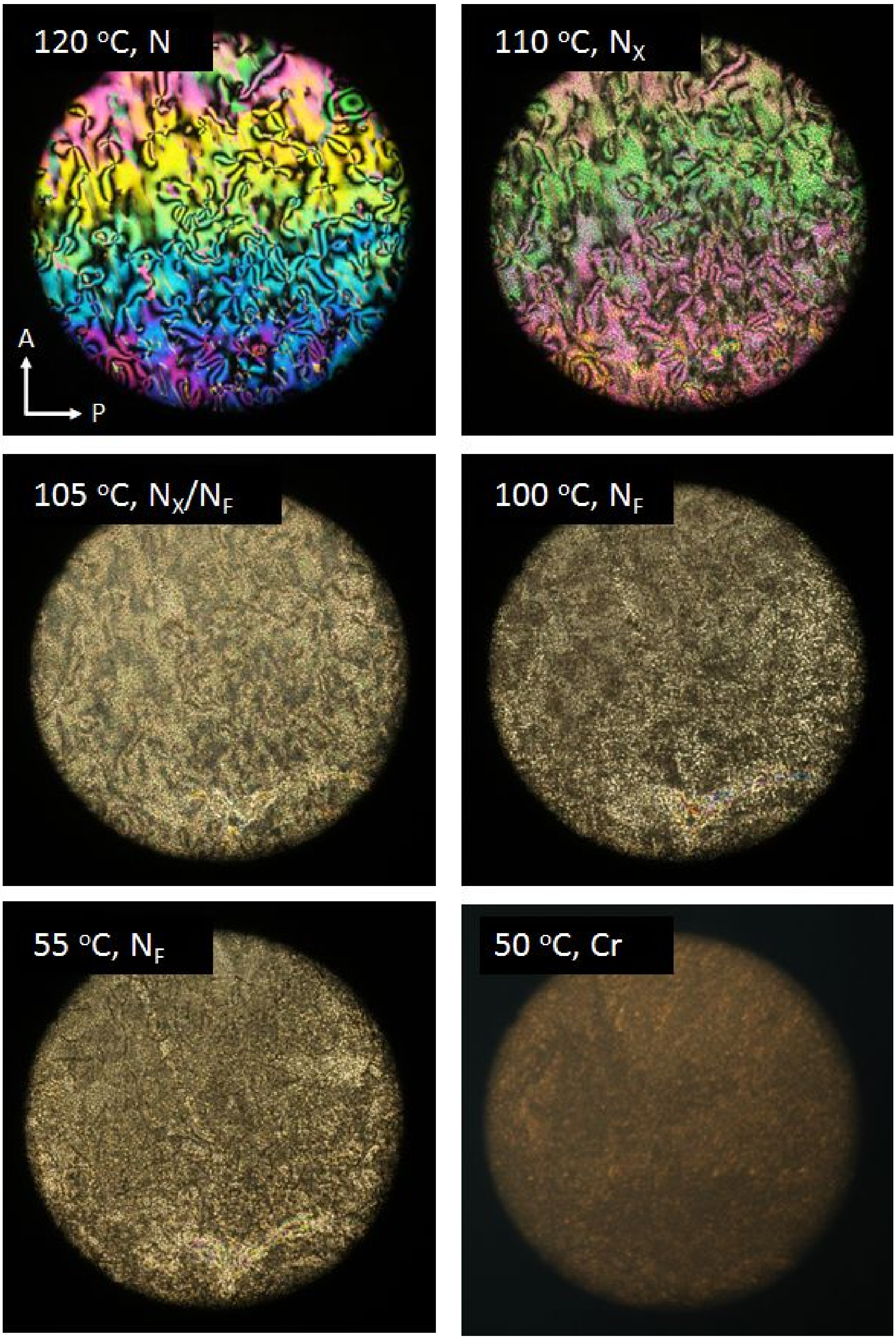

Figure S10 POM images of **EST-3** in the cooling run. **EST-3** was introduced in the cell composed of planer orientation glass substrates and then POM observation was performed.

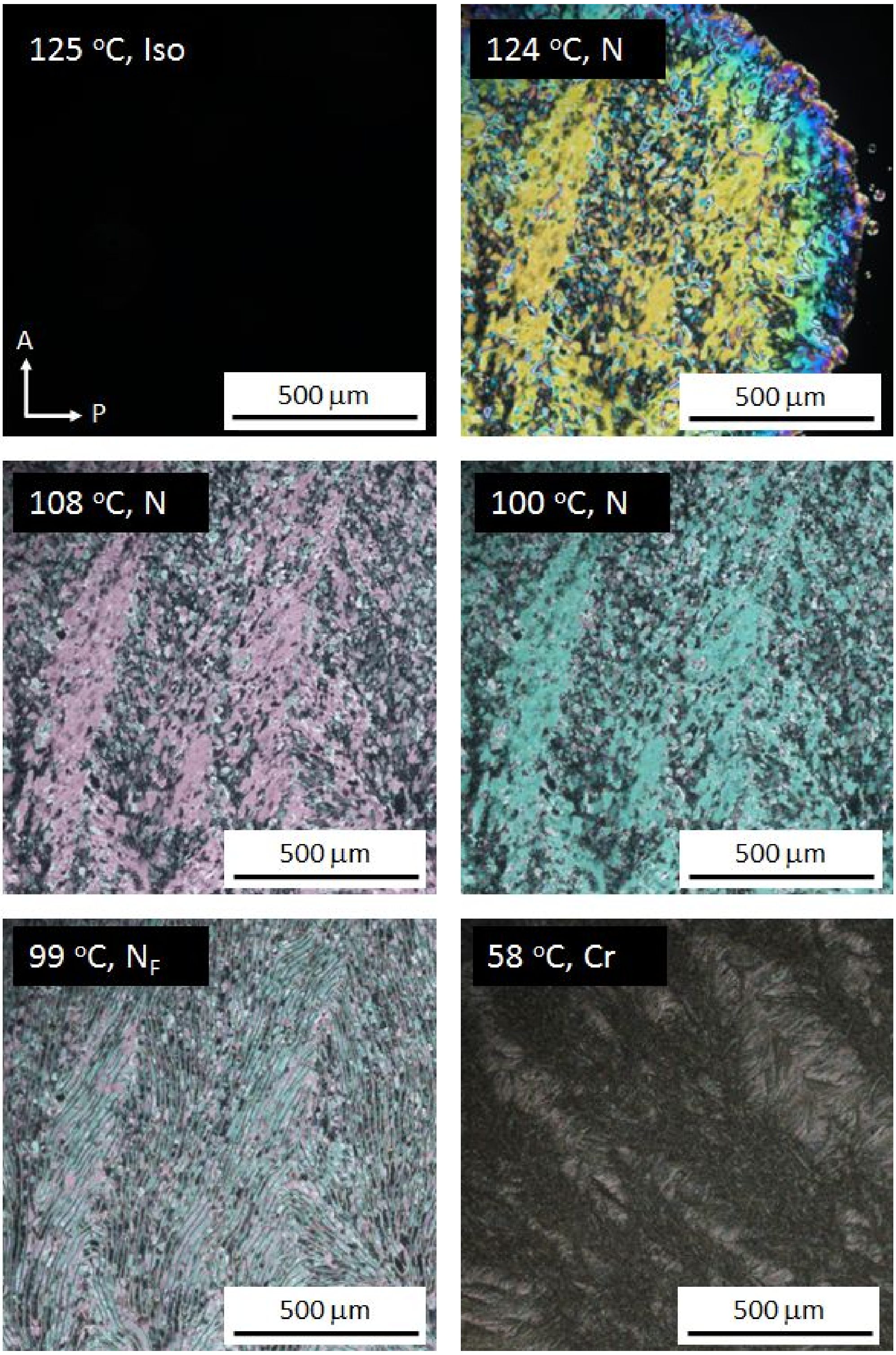

Figure S11 POM images of **EST-3** in the cooling run. **EST-3** was injected into the cell composed of non -treated glass substrates and then POM observation was performed.

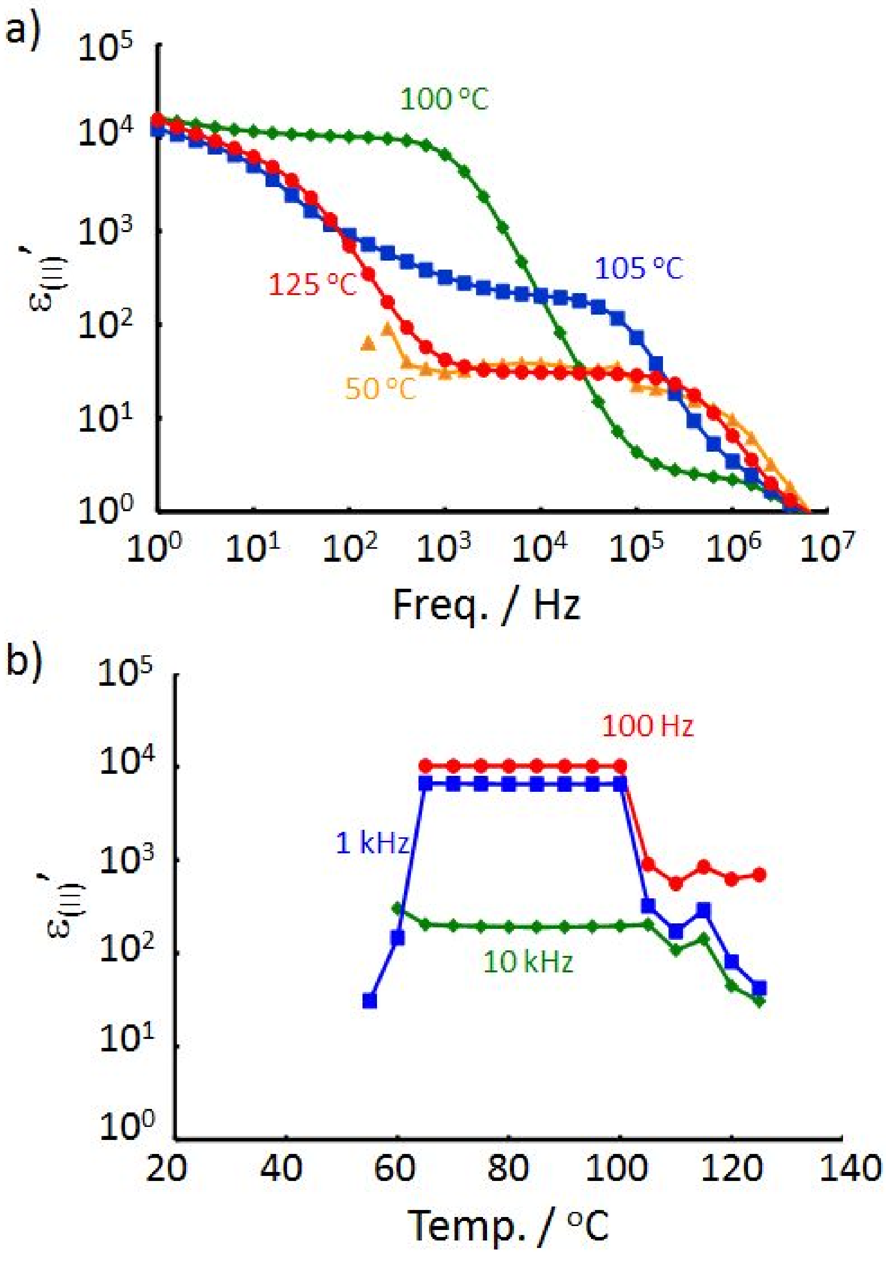

Figure S12 a) Frequency dependences of dielectric constant of **EST-3** in the cooling run. b) Temperature dependences of dielectric constant at different frequencies. **EST-3** was inserted in the cell consisting in homeotropically oriented glass substrates and then the measurements were carried out.

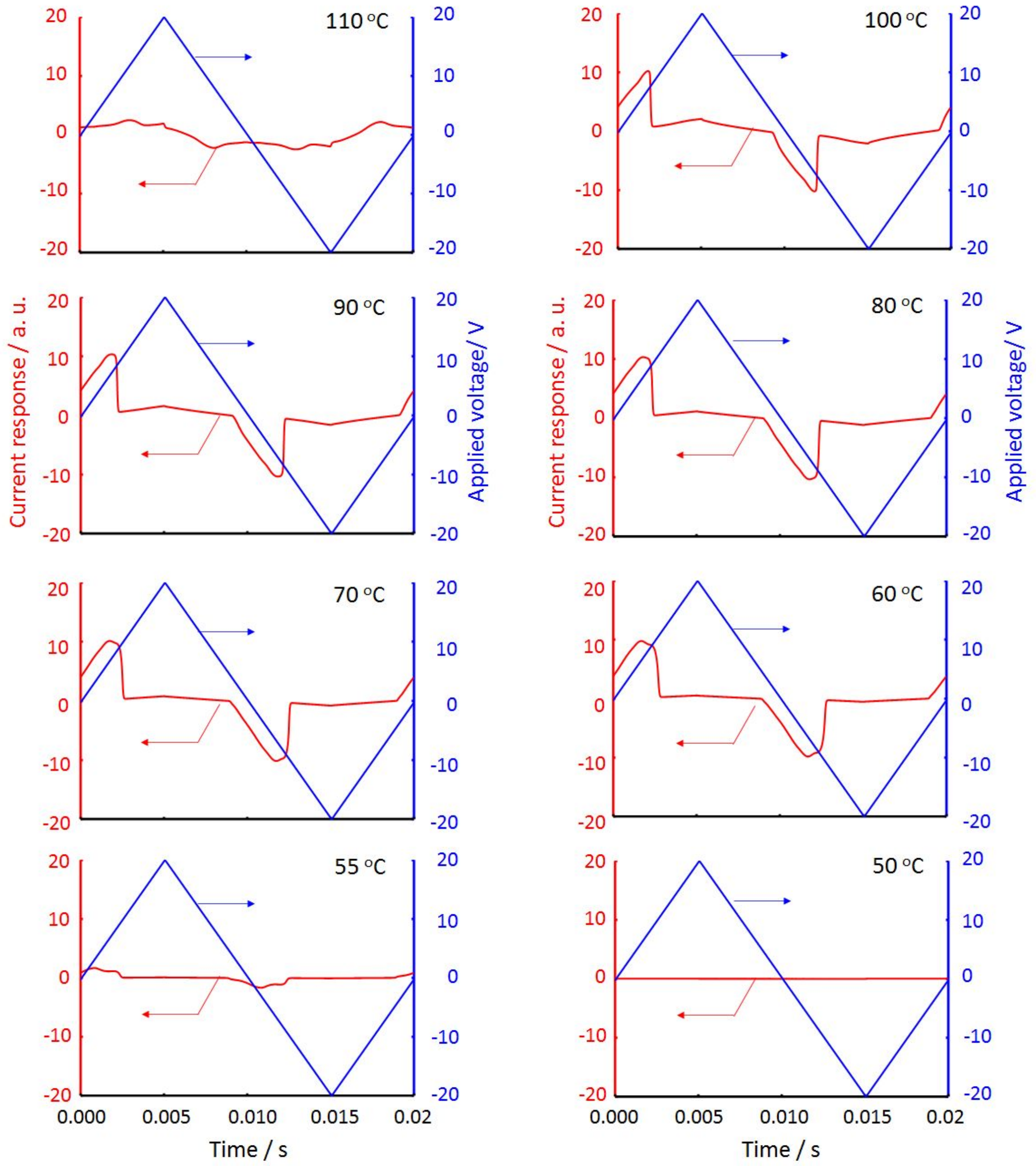

Figure S13 Switching current response of **EST-3** under an applied triangular wave voltage in the cooling run. $V_{P–P}$ = 40 V. *f* = 100 Hz. Red and black lines denote current response and applied triangular wave voltage, respectively.

5. Characterization of EST-4

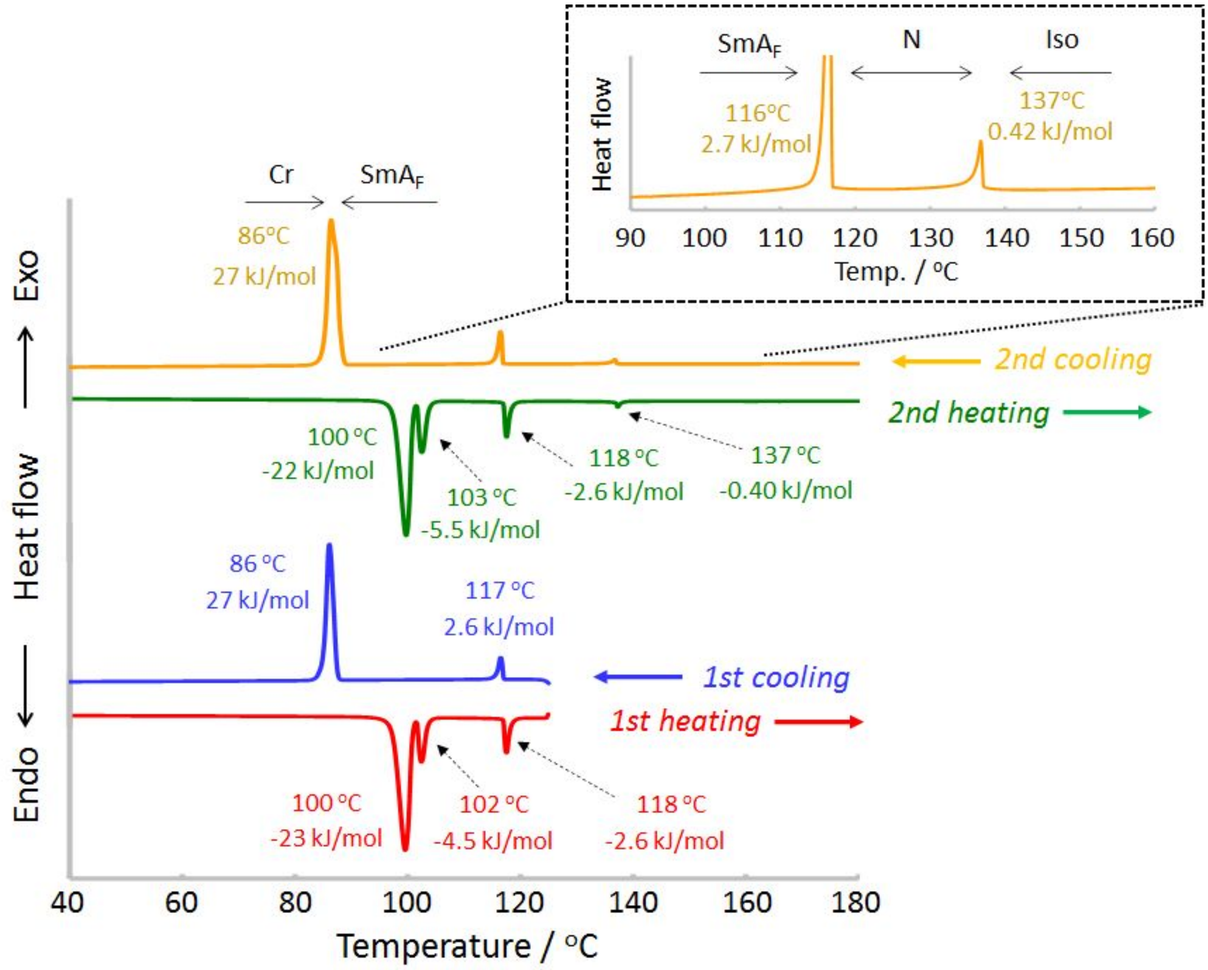

Figure S14 DSC charts of **EST-4**. Rate: 5 °C min^{-1}. The attribution of phase transitions in the 2nd cooling run is shown. The magnified profile with the identification of phase transitions in the 2nd cooling run is shown.

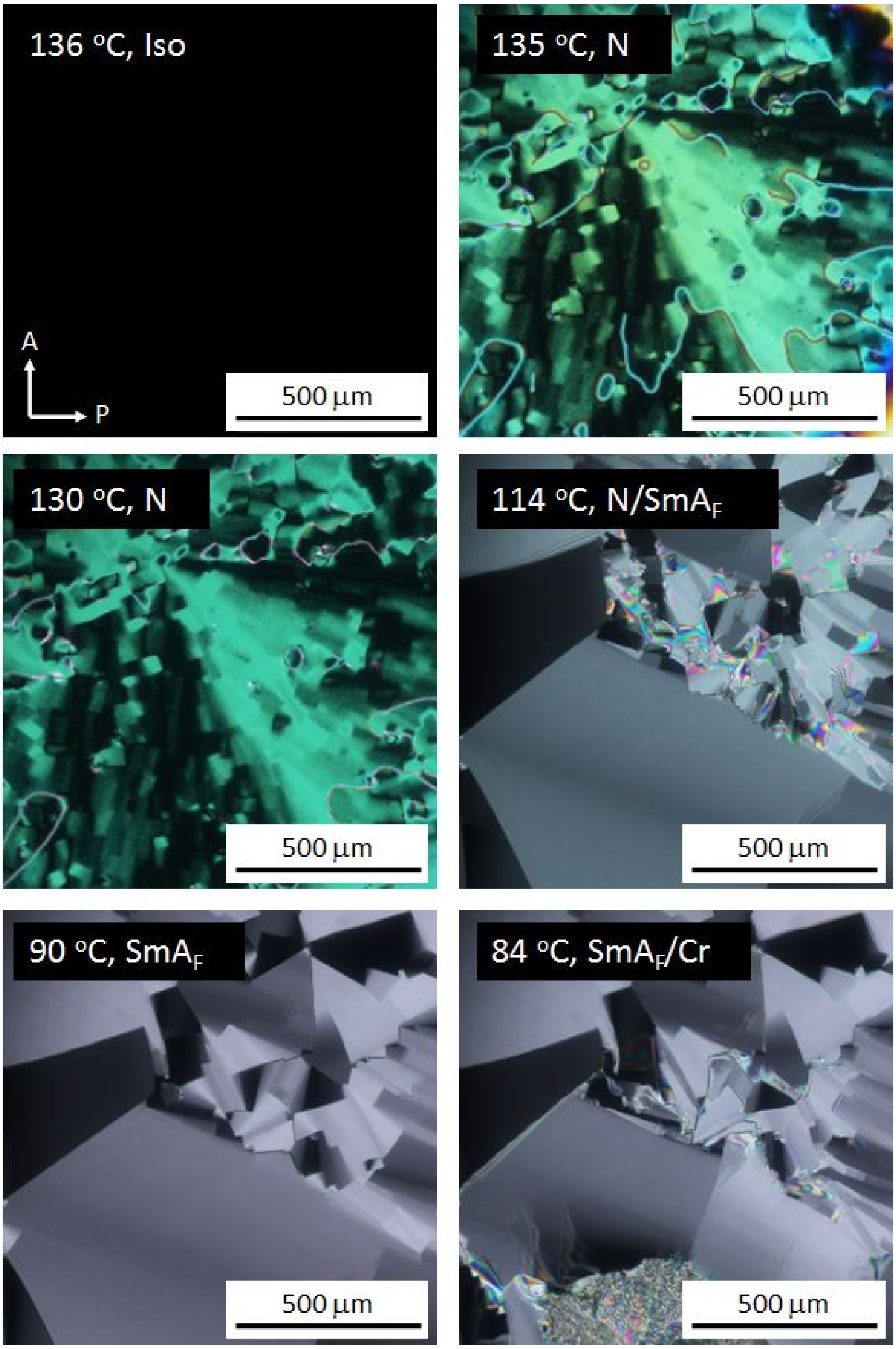

Figure S15 POM images of **EST-4** in the cooling run. **EST-4** was injected into the cell composed of non -treated glass substrates and then POM observation was performed. In POM images at 130 and 135 °C, a cybotactic N-like texture is partially observed.

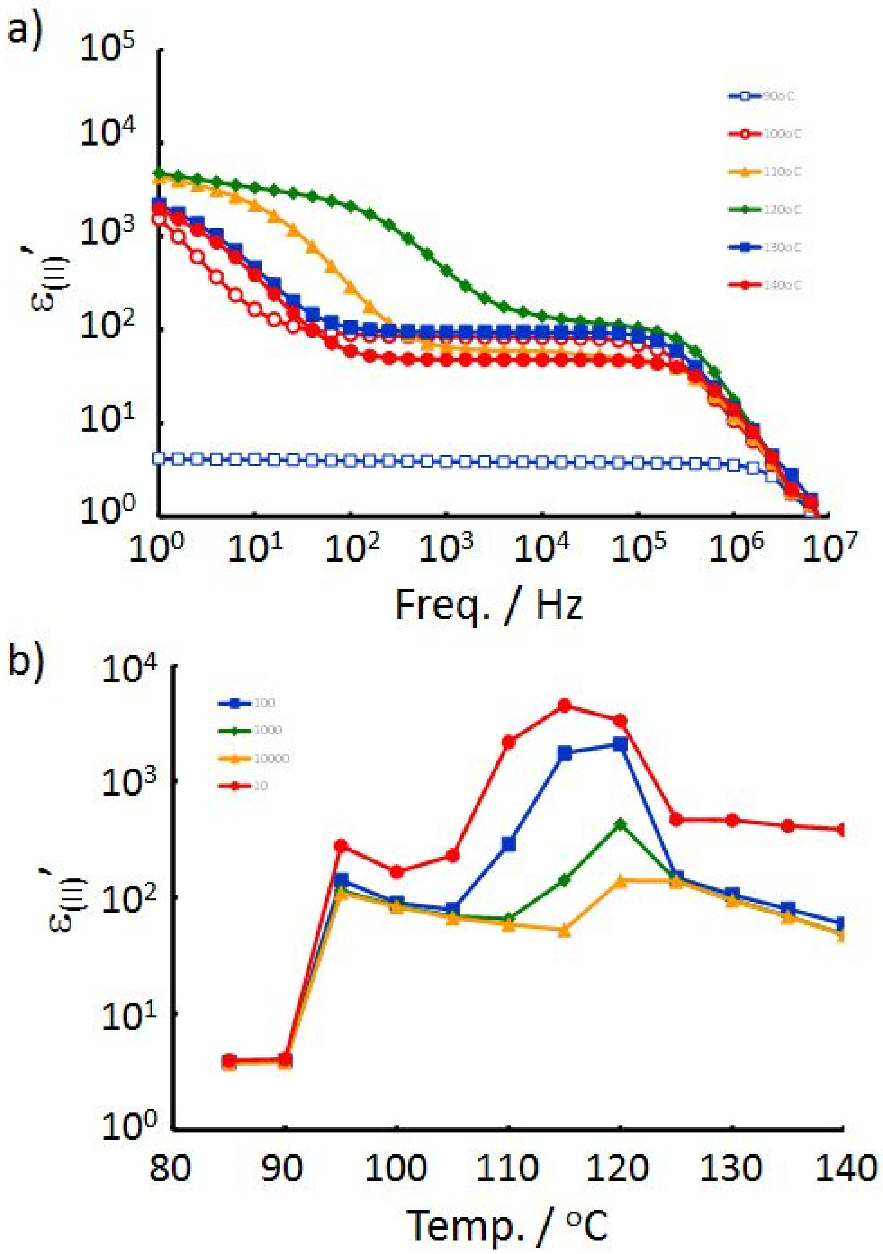

Figure S16 a) Frequency dependences of dielectric constant of **EST-4** in the 1st cooling run. b) Temperature dependences of dielectric constant at different frequencies. **EST-4** was inserted in the cell consisting in homeotropically oriented glass substrates and then the measurements were carried out.

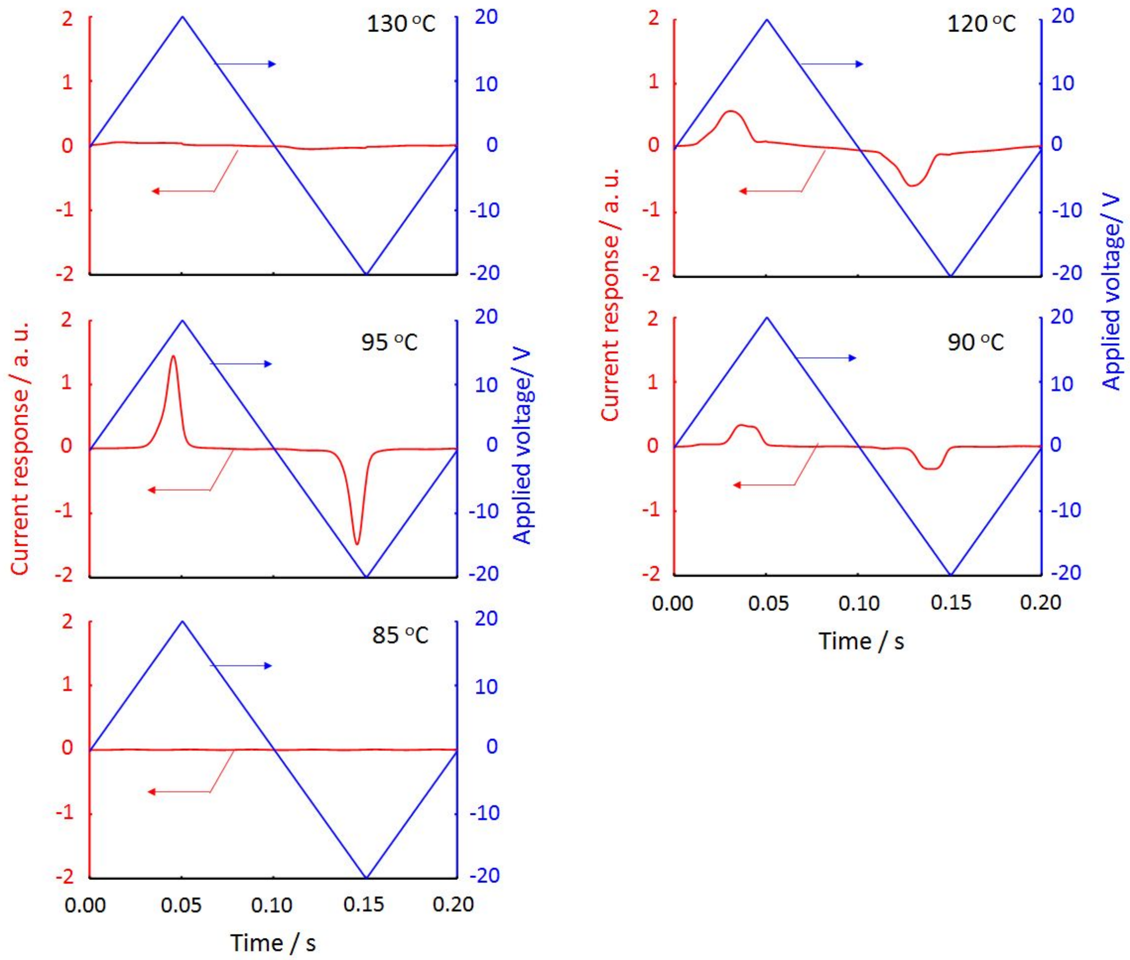

Figure S17 Switching current response of **EST-4** under an applied triangular wave voltage in the cooling run. $V_{P\text{–}P}$ = 40 V. *f* = 10 Hz. Red and black lines denote current response and applied triangular wave voltage, respectively.

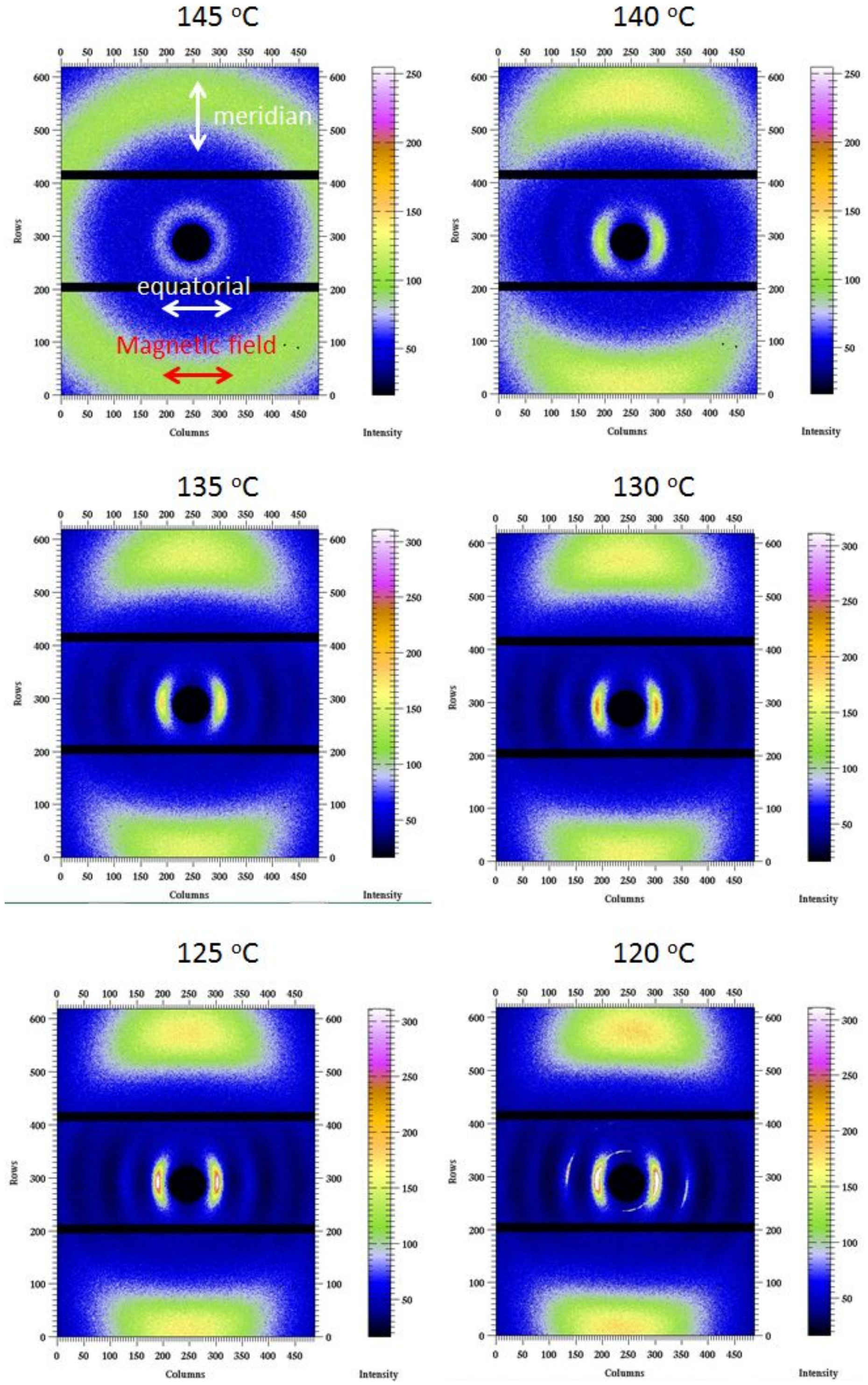

Figure 18 2-D SAXS pattern images of **EST-4** in the cooling run from 145 to 120 °C. Wavelength of X ray: 0.12 nm. A magnetic field (approximately 560 mT) is applied in the equatorial direction.

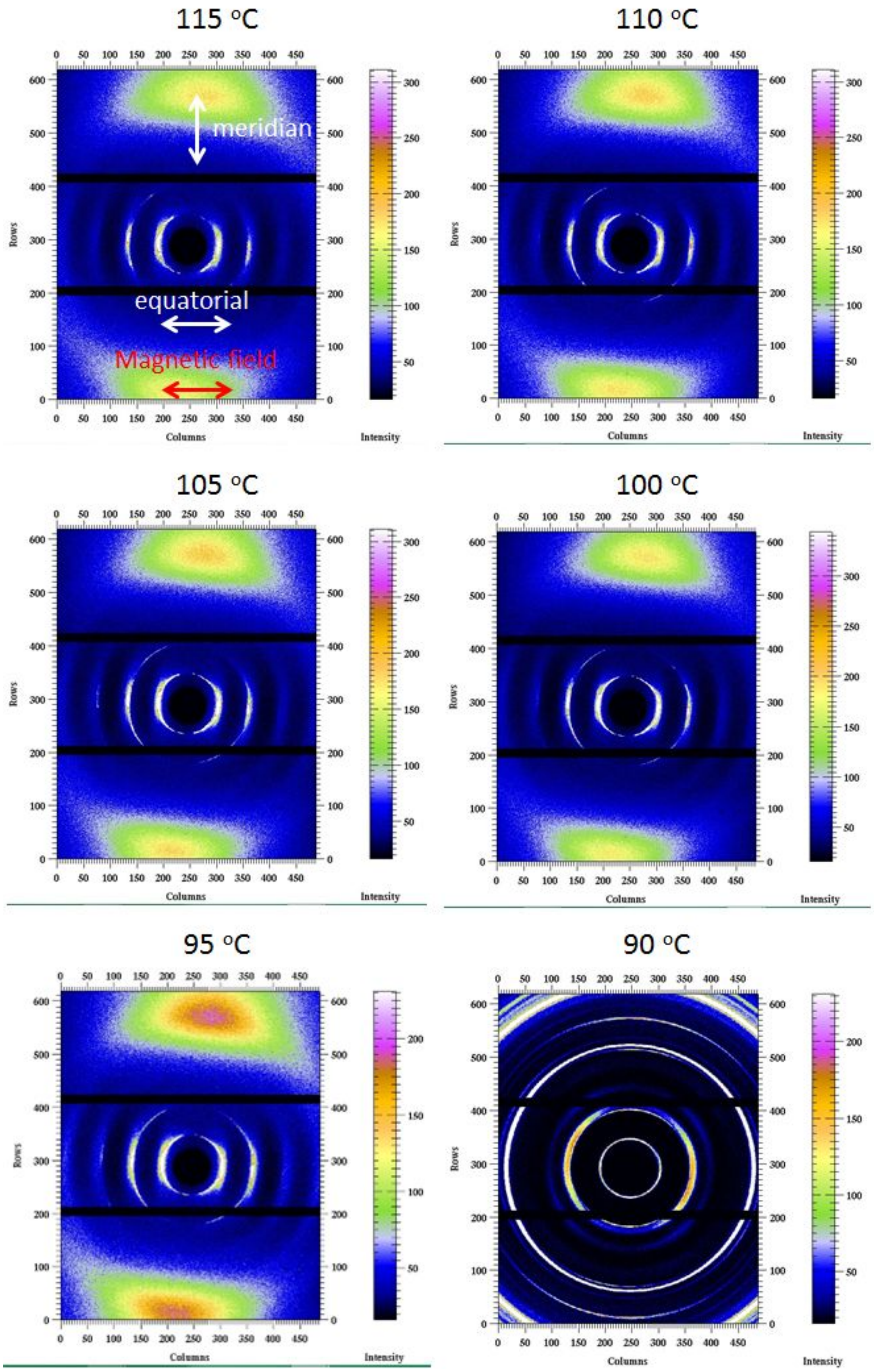

Figure 19 2-D SAXS pattern images of **EST-4** in the cooling run from 115 to 90 °C. Wavelength of X-ray: 0.12 nm. A magnetic field (approximately 560 mT) is applied in the equatorial direction.

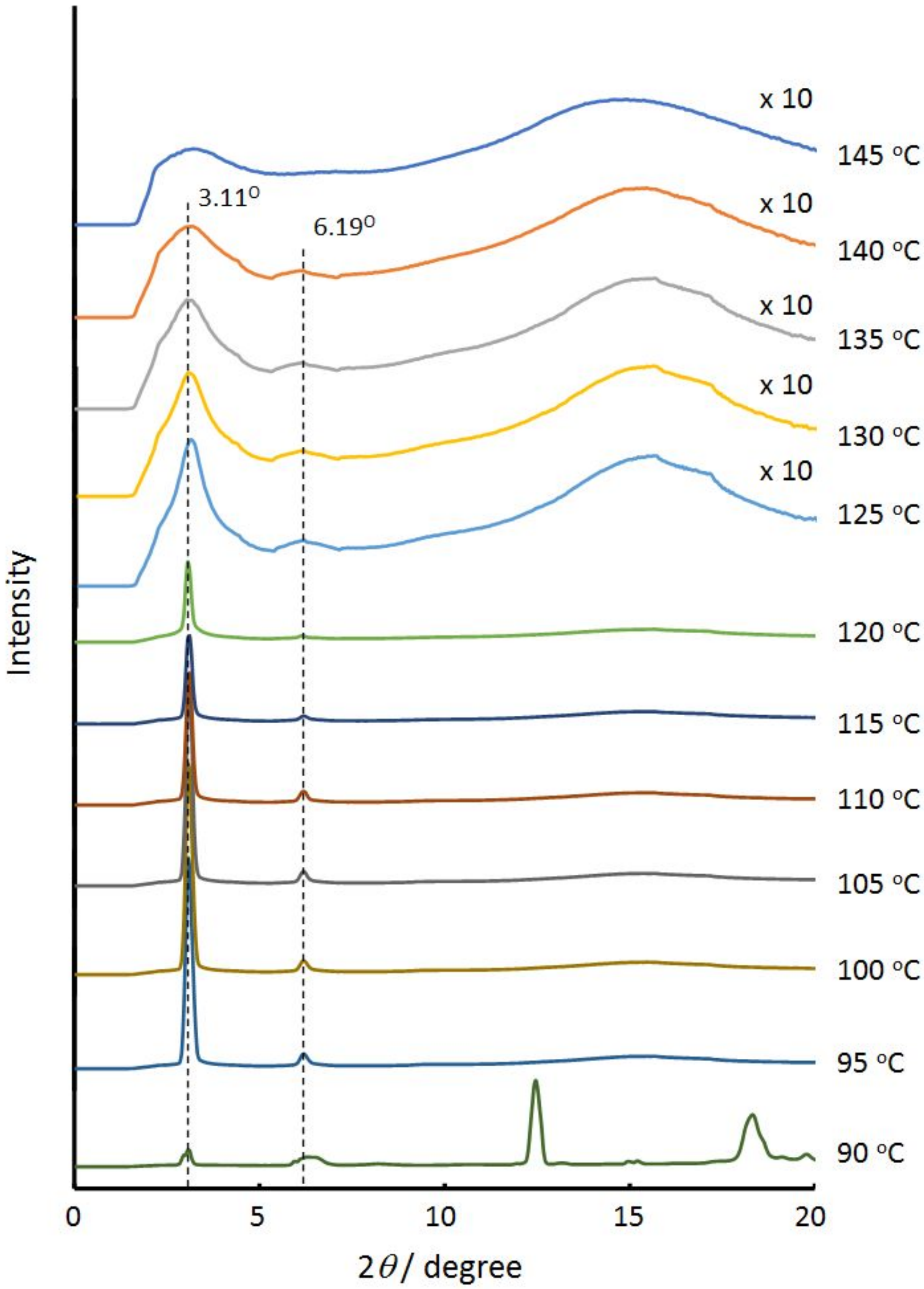

Figure 20 1-D SAXS profiles of **EST-4** in the cooling run obtained from 2D XRD images. λ = 0.12 nm. The magnified profiles (×10) at 125–145 °C are shown.

References

(1) Kikuchi, H.; Matsukizono, H.; Iwamatsu, K.; Endo, S.; Anan, S.; Okumura, Y. Fluid Layered Ferroelectrics with Global $C_{\infty v}$ Symmetry. *Adv. Sci.* **2022**, *9*, 2202048.

(2) Ju, S-P.; Huang, S.-C.; Lin, K.-H.; Chen, H.-Y.; Shen, T.-K. Prediction of Optical and Dielectric Properties of 4‑Cyano-4-pentylbiphenyl Liquid Crystals by Molecular Dynamics Simulation, Coarse-Grained Dynamics Simulation, and Density Functional Theory Calculation. *J. Phys. Chem. C* **2016**, *120*, 14277-14288.

(3) Gaussian 16, Revision C.01, Frisch, M. J.; Trucks, G. W.; Schlegel, H. B.; Scuseria, G. E.; Robb, M. A.; Cheeseman, J. R.; Scalmani, G.; Barone, V.; Petersson, G. A.; Nakatsuji, H.; Li, X.; Caricato, M.; Marenich, A. V.; Bloino, J.; Janesko, B. G.; Gomperts, R.; Mennucci, B.; Hratchian, H. P.; Ortiz, J. V.; Izmaylov, A. F.; Sonnenberg, J. L.; Williams-Young, D.; Ding, F.; Lipparini, F.; Egidi, F.; Goings, J.; Peng, B.; Petrone, A.; Henderson, T.; Ranasinghe, D.; Zakrzewski, V. G.; Gao, J.; Rega, N.; Zheng, G.; Liang, W.; Hada, M.; Ehara, M.; Toyota, K.; Fukuda, R.; Hasegawa, J.; Ishida, M.; Nakajima, T.; Honda, Y.; Kitao, O.; Nakai, H.; Vreven, T.; Throssell, K.; Montgomery, J. A., Jr.; Peralta, J. E.; Ogliaro, F.; Bearpark, M. J.; Heyd, J. J.; Brothers, E. N.; Kudin, K. N.; Staroverov, V. N.; Keith, T. A.; Kobayashi, R.; Normand, J.; Raghavachari, K.; Rendell, A. P.; Burant, J. C.; Iyengar, S. S.; Tomasi, J.; Cossi, M.; Millam, J. M.; Klene, M.; Adamo, C.; Cammi, R.; Ochterski, J. W.; Martin, R. L.; Morokuma, K.; Farkas, O.; Foresman, J. B.; Fox, D. J. Gaussian, Inc., Wallingford CT, **2016**.
